# Two distinct modes of Vgll4-mediated Tead regulation control organ size in zebrafish

**DOI:** 10.1038/s42003-026-10098-y

**Published:** 2026-04-25

**Authors:** Alicia Lardennois, Veronika Duda, Chaitanya Dingare, Petra A. Klemmt, Constanze Heinzen, Lucas Desruelles, Melanie Heyde, David Simon Kleinhans, Thorsten Falk, Carsten Schelmbauer, Olivia Mozolewska, Sofia Papadopoulou, Jason J. K. Lai, Didier Y. R. Stainier, Virginie Lecaudey

**Affiliations:** 1 Department of Developmental Biology of Vertebrates, Institute of Cell Biology and Neuroscience, Goethe University Frankfurt am Main, Frankfurt am Main, Germany; 2 Image Analysis Lab, Institute for Computer Science, University of Freiburg, Freiburg, Germany; 3 Department of Developmental Genetics, Max Planck Institute for Heart and Lung Research, Bad Nauheim, Germany; 4 Present Address: Laboratory of Comparative Developmental Dynamics, Department of Genetics, University of Cambridge, Cambridge, UK

**Keywords:** Organogenesis, HIPPO signalling

## Abstract

Control of organ size during development and homeostasis relies on balanced regulation of Hippo pathway transcriptional output, yet how TEAD activity is precisely regulated in vivo remains unclear. Using the zebrafish posterior lateral line (pLL) we show that Yap1 is required early in pLL progenitors to ensure sufficient cell numbers in the migrating primordium. In contrast, the two zebrafish Vgll4 paralogs, Vgll4b and Vgll4l, act partially redundantly to limit pLLP size and cell number. Through loss- and gain-of-function analyses, epistasis experiments, transcriptional reporter quantification and pharmacological treatments, we find that Vgll4 restricts Tead-dependent transcription through two co-existing mechanisms: inhibition of Yap1–Tead–mediated transcriptional activation and Tead-dependent repression. Together, our findings reconcile the competitive and default repression models of VGLL4 function and provide an integrated framework for how VGLL4 fine-tunes TEAD output to control tissue growth in vivo.

## Abstract (web summary)

Genetic and functional analysis in zebrafish shows that Vgll4 limits tissue growth by inhibiting Yap1–Tead– mediated activation and promoting Tead-dependent repression, providing a framework for how Hippo pathway output regulates organ size in vivo.

## Introduction

During embryonic development, organs must grow and acquire the correct size and shape. While cell proliferation is essential for organ formation, its dysregulation can lead to excessive growth and tumor formation. Therefore, proliferation must be precisely controlled and coordinated with other developmental processes to ensure that organs reach their proper size without exceeding it. Yet, the precise mechanisms underlying this regulation remain only partially understood.

The Hippo signaling pathway has emerged as a key regulator of organ size during development and homeostasis. Initially identified in *Drosophila*^1–4^, the Hippo pathway is highly conserved in vertebrates^5–7^. The Hippo pathway consists of a core kinase cascade, which phosphorylates the downstream effectors YAP1 (Yes-associated protein 1) and its homolog WWTR1 (WW domain-containing transcription regulator 1, also known as TAZ), leading to their cytoplasmic sequestration or degradation. Conversely, when Hippo signaling is inactive, YAP1/WWTR1 translocates to the nucleus, where they drive the transcription of genes that promote proliferation and suppress apoptosis^8–13^. The Hippo pathway includes various upstream regulators, often membrane-associated cell junction proteins, that influence YAP1/WWTR1 localization and stability. In addition, YAP1 activity can also be modulated through Hippo-independent routes. For example, Src family kinases directly phosphorylate YAP1 on specific residues, promoting its nuclear localization and transcriptional activity independently of LATS-mediated control^14–16^. Moreover, mechanical cues such as substrate stiffness, cytoskeletal tension, and nuclear deformation can drive YAP1 nuclear import via importin-mediated transport, bypassing Hippo kinase regulation^17,18^. These alternative modes of regulation highlight the integration of biochemical and mechanical inputs in controlling YAP1 function.

While the control of YAP1 activity by membrane-associated and cytosolic proteins has been well-studied, its nuclear regulation is less understood. YAP1 is a transcription cofactor that lacks a DNA-binding domain. It must interact with DNA-binding proteins, particularly TEAD-family transcription factors, to regulate target genes expression. TEAD proteins also interact with Vestigial-like family member 4 (VGLL4), which functions as a tumor suppressor^19–21^. Several studies have shown that VGLL4 and YAP1 compete with each other because both bind to the same region of TEAD^19,22,23^. This competition has been proposed to determine the outcome of gene expression. Elevated levels of VGLL4 competitively displace YAP1, leading to the downregulation of genes associated with cell proliferation and survival, accounting for the VGLL4 tumor-suppressive activity^24,25^. Conversely, when YAP1 predominates over VGLL4 and binds to TEAD, it activates genes promoting cell proliferation and survival. In this scenario, the balance is shifted towards promoting cell growth and potentially contributing to tumor development^19,21^. In addition, VGLL4 and its Drosophila homolog Tgi have been shown to function as default repressors together with TEAD/Sd, repressing Hippo target genes in the absence of YAP1/Yki^23,26,27^. In this context, it has been proposed that the primary function of YAP1/Yki, during normal tissue growth, is to relieve the VGLL4/Tgi-mediated default repression^23,26,27^. Yet, it remains unclear, whether VGLL4 might not also limit organ growth during development by competing with YAP1 transcriptional activity in a context-dependent manner.

The zebrafish posterior lateral line primordium (pLLP) offers a simple and robust system to investigate this question. As it delaminates from the pLL placode, the pLLP contains approximately 120 epithelial cells that migrate as a group beneath the skin and deposit neuromasts at their trailing end^28–30^. Its compact size, robust cell number, and superficial position make the pLLP an ideal system for high-resolution imaging, allowing automatic quantification of cell number, shapes, and volumes. Combined with the amenability of zebrafish embryos to genetic and pharmacological approaches, the model has clear advantages for dissecting the molecular mechanisms controlling epithelial tissue growth. On the one hand, the canonical Wnt signaling pathway, via Lef1, promotes proliferation in the leading region, thus partially compensating for neuromast deposition^31–35^. On the other hand, we previously reported that Amotl2a, a Motin family protein, limits the number of pLLP cells by inhibiting Yap1 and Lef1 activity^36^.

Here, we investigated the nuclear mode of action of Yap1 and its regulation by Vgll4 using the zebrafish pLLP as an in vivo model. We demonstrate that the growth-promoting activity of Yap1 is Tead-dependent and is required early, before pLL placode formation. By combining gain- and loss-of-function, rescue and epistasis experiments with quantitative imaging and reporter analyses, we show that *vgll4b* and *vgll4l*, the two zebrafish *vgll4* paralogs expressed in the pLLP, function partially redundantly to limit pLLP size and cell number. Our results indicate that the tumor-suppressor function of Vgll4 in the pLLP operates through two Tead-dependent mechanisms: Tead-dependent repression co-existing with competition that sterically hinders Yap1-Tead complex formation and transcriptional activation. Our findings thus reconcile the two main conceptual models of VGLL4 function: the default repressor and the YAP1/TEAD-competitor model, demonstrating that both can co-exist within the same tissue to control normal organ growth in vivo.

## Results

### The growth-promoting activity of Yap1 in the pLLP depends on Tead proteins

**Fig. 1:**
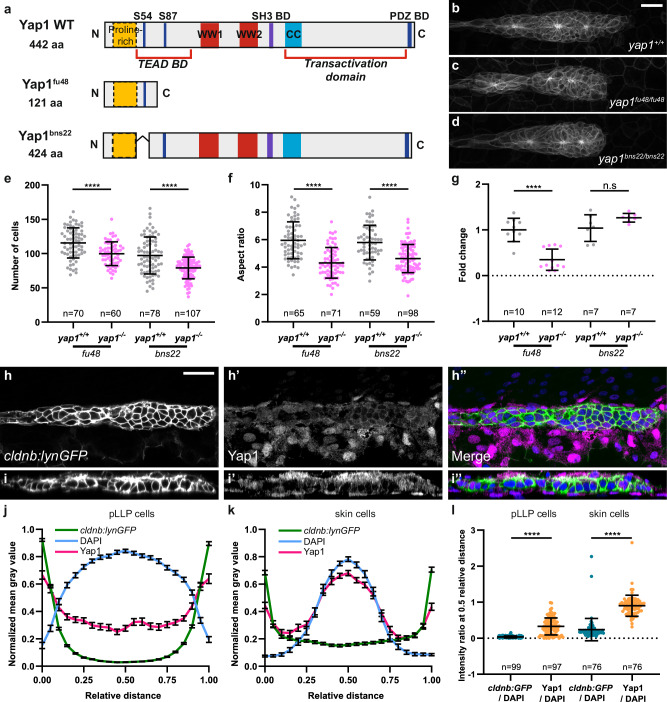
Tead-binding is essential for Yap1-mediated proliferation in the pLLP. **a** Schematic representation of the WT Yap1 and the mutant Yap1^fu48^ and Yap1^bns22^ proteins. Maximum intensity projection (MIP) of spinning disc confocal Z-stacks showing the pLLP in WT (**b**), *yap1*^*fu48*^ (**c**), and *yap1*^*bns22*^ (**d**) homozygous mutant embryos. Quantification of cell number (**e**) and aspect ratio (**f**) of the pLLP across genotypes (*N* = 3 independent replicates). **g** Relative *yap1* transcript levels measured by qPCR in *yap1*^*+/+*^ and *yap1*^*−/−*^ siblings carrying the *fu48* or *bns22* alleles at 32 hpf (*N* = 2 independent replicates). Single Z plan of confocal Z-stacks (**h**–**h”**) and orthogonal views (**i**–**i”**) showing Yap1 localization in 32 hpf WT embryos. Plot profile intensity across pLLP cells (**j**) and neighboring skin cells (**k**) in *cldnb:lynGFP* embryos at 32 hpf. Normalized mean gray values are shown for *cldnb:lynGFP* (membranes, green), DAPI (nuclei, blue), and Yap1 (magenta). **l** Quantification of normalized Yap1 and *cldnb:lynGFP* intensities relative to DAPI at 0.5 relative distance (*N* = 1 independent replicate). Unless otherwise indicated, the pLLP is marked by *cldnb:lynGFP* in all panels (**b**–**d**, **h**–**i”**). Data are presented as mean ± SD, except for (**j**, **k**), data are mean ± SEM. Unpaired *t*-tests (Mann–Whitney) were conducted. In all figures MIP are maximum intensity projections of spinning disc confocal Z-stacks. Scale bars: 20 μm (**b**–**d**, **h**–**i”**).

To test whether Yap1’s growth-promoting function in the pLLP depends on Tead transcription factors, we used *yap1*^*bns22*^, a *yap1* allele encoding a Yap1 protein lacking 18 amino acids—including S54—in the predicted Tead-binding domain (Fig. 1a)^37–40^. We quantified the size and shape of the pLLP in *yap1*^*bns22*^ mutant and compared them to those in *yap1*^*fu48*^, an allele carrying an early premature STOP codon (Fig. 1a)^36^. Both *yap1*^*fu48*^ and *yap1*^*bns22*^ mutant pLLP appeared smaller and rounder (Fig. 1b–d), their total volume was reduced (Supplementary Fig. 1a), as did their cell count by approximately 20% compared to siblings (Fig. 1e). The aspect ratio was also similarly reduced in both alleles (Fig. 1f). The number of rosettes was decreased, while the parameter quantifying rosette shape and quality (rosettiness—see “Materials and methods”), remained unaffected (Supplementary Fig. 1b, c). This suggests that *yap1* mutant primordia form normal rosettes, but fewer of them.

To ensure that we were specifically testing the requirement for Tead binding, we confirmed that the mutant Yap1^bns22^ protein was effectively produced and stable. qRT-PCR experiments showed that while *yap1*^*fu48*^ mutant mRNA levels were significantly reduced (65%), *yap1*^*bns22*^ mutant mRNA levels remained unchanged (Fig. 1g). This suggests that *yap1*^*fu48*^ mRNAs, but not *yap1*^*bns22*^, are degraded, most likely by nonsense-mediated mRNA decay (NMD)^41^. To measure Yap1 protein levels, we generated an antibody against the zebrafish protein (VL7801)^42,43^. Yap1 was present in pLLP cells of wild-type (WT) embryos at 32 hours post-fertilization (hpf). While neighboring somitic cells showed distinct nuclear localization, Yap1 localized more in the cytoplasm of pLLP cells (Fig. 1h–i”). Fluorescence intensity profiles further confirmed these differences, showing reduced overlap between Yap1 and DAPI signals in pLLP cells compared with skin cells (Fig. 1j, k). Quantification of normalized intensities in the center of each cell (0.5 relative distance) revealed significantly higher nuclear Yap1 enrichment in skin cells than in pLLP cells, consistent with predominantly cytoplasmic Yap1 localization in the pLLP (Fig. 1l). No signal was detected in *yap1*^*fu48/fu48*^ (Supplementary Fig. 1f–f”) mutants, confirming the specificity of the antibody and suggesting these are null mutants. In contrast, Yap1 proteins were still present in *yap1*^*bns22/bns22*^ mutants, at levels equivalent to WT Yap1. The signal was more diffuse in *yap1*^*bns22/bns22*^ mutants, suggesting a shift of Yap1^bns22^ mutant protein towards the cytoplasm (Supplementary Fig. 1i–m), consistent with published data showing that binding to TEAD retains YAP1 in the nucleus^44^. Wwtr1 protein was also detected in the pLLP. Similar to Yap1, the signal was rather cytoplasmic, except in some cells at the pLLP periphery, in which the signal was both cytoplasmic and nuclear (Supplementary Fig. 1g–g”). Loss of signal in *wwtr1*^*fu55/fu55*^ mutants confirmed the antibody specificity (Supplementary Fig. 1h–h”). *wwtr1* mutants showed no obvious phenotype on pLLP cell count, indicating that although Wwtr1 is expressed in the pLLP, it is not required alone to regulate its size and shape (Supplementary Fig. 1d). These results indicate that Yap1, but not Wwtr1, is essential for the pLLP to acquire a normal size and shape, and that this activity depends on its interaction with Tead proteins.

**Fig. 2:**
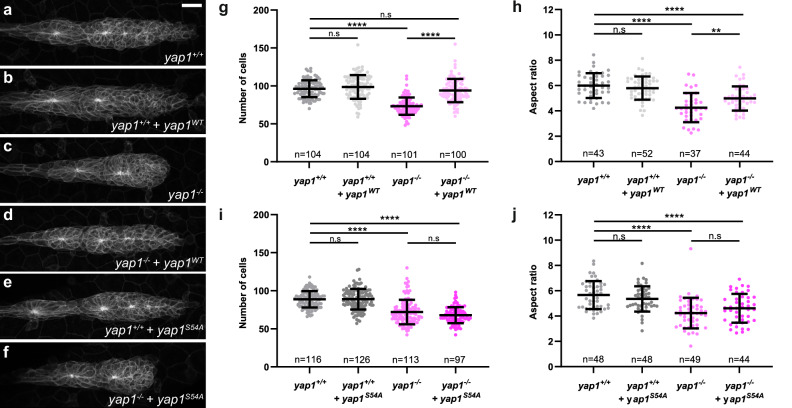
A Tead-Binding-Deficient Yap1 mutant fails to rescue *yap1* loss-of-function phenotype. MIP of confocal Z-stacks showing differences in pLLP size in uninjected *yap1*^*+/+*^ embryos (**a**), *yap1*^*+/+*^ embryos injected with *yap1*-WT mRNA (**b**), uninjected *yap1*^*−/−*^ embryos (**c**), *yap1*^*−/−*^ embryos injected with *yap1*-WT mRNA (**d**), *yap1*^*+/+*^ embryos injected with *yap1-S54A* mRNA (**e**), *yap1*^*−/−*^ embryos injected with *yap1-S54A* mRNA (**f**). Quantification of pLLP cell number (**g**, **i**) and aspect ratio (**h**, **j**) of the pLLP in the indicated conditions (*N* = 3 independent replicates). Data are presented as mean ± SD. Unpaired *t*-tests (Mann–Whitney) were conducted. Scale bar: 20 μm.

To confirm this, we attempted to rescue the *yap1* mutant phenotype using WT Yap1 or Yap1^S54A^, a Tead-binding defective form of Yap1^40^. While *yap1*^*WT*^ mRNA fully rescued both cell number and aspect ratio to control levels in *MZyap1*^*fu48/fu48*^ embryos (Fig. 2a, c, d, g, h), *yap1*^*S54A*^ mRNA did not rescue either (Fig. 2a, c, f, i, j). Neither *yap1*^*WT*^ nor *yap1*^*S54A*^ overexpression in WT induced any phenotype or altered cell numbers in the pLLP (Fig. 2a, b, e, g, i). Similar results were obtained when rescuing the *yap1*^*bns22*^ mutant (Supplementary Fig. 2a–f). Altogether, these results indicate that Yap1 physically interacts with Tead to maintain a proper number of cells in the pLLP.

### Vgll4b and Vgll4l are required to limit the number of cells in the pLLP

**Fig. 3:**
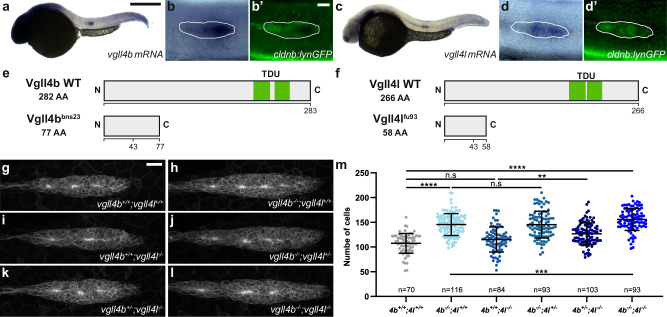
Vgll4b and Vgll4l are required to limit the number of cells in the pLLP. Overview (**a**, **c**) and higher magnification images (**b**, **b’**, **d**, **d’**) taken on a brightfield microscope of 32 hpf *cldnb:lynGFP* embryos stained by in situ hybridization (ISH) for *vgll4b* (**a**, **b**) and *vgll4l* (**c**, **d**) and counterstained with an anti-GFP antibody (**b’**, **d’**). Schematic representation of WT Vgll4b and the mutant form Vgll4b^bns23^ (**e**), and WT Vgll4l and the mutant form Vgll4l^fu93^ (**f**). MIP of confocal Z-stacks showing the pLLP in *vgll4b*^*+/+*^*;vgll4l*^*+/+*^ (**g**), *vgll4b*^*−/−*^*;vgll4l*^*+/+*^ (**h**), *vgll4b*^*+/+*^*;vgll4l*^*−/−*^ (**i**), *vgll4b*^*−/−*^*;vgll4l*^*+/−*^ (**j**), *vgll4b*^*+/−*^*;vgll4l*^*−/−*^ (**k**), *vgll4b*^*−/−*^*;vgll4l*^*−/−*^ (**l**) embryos. **m** Quantifications of pLLP cell number for each indicated group (*N* ≥ 4 independent replicates). Data are presented as mean ± SD. Unpaired *t*-tests (Mann–Whitney) were conducted. Scale bar: 400 μm (**a**, **c**), 20 μm (**b**–**d’**, **g**–**l**).

Vestigial-like family member 4 (VGLL4) has been identified in *Drosophila* (Tgi) and in mammals as a transcriptional cofactor competing with YAP1 for binding to TEAD transcription factors^23,26^. To investigate whether Vgll4 proteins similarly compete with Yap1 in the pLLP, we first assessed the expression patterns of the three zebrafish *vgll4* paralogs^45^. *vgll4b* and *vgll4l* were expressed in the pLLP at 30 hpf (Fig. 3a–d’) and until the end of migration^45^. In contrast, *vgll4a* was not expressed in the pLLP (Supplementary Fig. 3c–c”). To test if *vgll4b* and *vgll4l* regulate pLLP cell count and size, we generated mutants for both genes. The recovered alleles carried deletions of 13 and 11 bp, respectively, leading to premature STOP codons (Fig. 3e, f, Supplementary Fig. 3a, b). *vgll4b* and *vgll4l* homozygous mutant embryos showed no obvious morphological defects, and adults were viable, consistent with recently published mutants of these genes^46,47^. While *vgll4l* embryos showed no phenotype on pLLP cell count, *vgll4b* embryos had significantly more pLLP cells than their control siblings, with a mean increase of 35% (Fig. 3g–i, m). To test whether NMD-mediated transcriptional adaptation may limit or mask the phenotype of *vgll4b* and *vgll4l* mutants, we quantified *vgll4b* and *vgll4l* mRNA levels in both mutants. *vgll4b* mRNA was reduced by 60% in *vgll4b* mutants, possibly due to NMD, while *vgll4l* mRNA levels were not increased (Supplementary Fig. 3g). In contrast, *vgll4l* mRNA levels were increased two-fold in *vgll4l* mutants, suggesting auto-inhibition without degradation. *vgll4b* mRNA levels were also not increased in *vgll4l* mutants (Supplementary Fig. 3h). This indicates that the loss of Vgll4b or Vgll4l activity is not compensated by an upregulation of *vgll4l* or *vgll4b* mRNA, respectively.

Since both genes are expressed in the pLLP, they could still functionally compensate for each other’s loss. To test this, we generated *vgll4b*;*vgll4l* double mutants. Not only was the number of cells in the *vgll4b*;*vgll4l* pLLP higher than in control siblings (mean increase of 50%, *p* < 0.0001), it was also significantly higher than in *vgll4b* single mutants (Fig. 3g–m). Quantification of the pLLP volumes showed the same trend as cell counts in both single and double mutants (Supplementary Fig. 3i), indicating that an increased cell count translates to an increase in organ size. No difference in the aspect ratio, or in the number or quality of rosettes was detected in any of the *vgll4* mutants (Supplementary Fig. 3j–l). The final neuromast pattern was not significantly affected, despite the increased pLLP cell number (Supplementary Fig. 3m–r). To rule out the possibility that *vgll4a* may compensate for vgll4b/l loss-of-function, we confirmed that *vgll4a* was not expressed in the double mutants using in situ hybridization (ISH) (Supplementary Fig. 3c–f”). These results show that Vgll4l and Vgll4b function largely redundantly and are required to limit the number of cells in the pLLP.

### Vgll4b reduces cell numbers in the pLLP and rescues Vgll4 activity loss

**Fig. 4:**
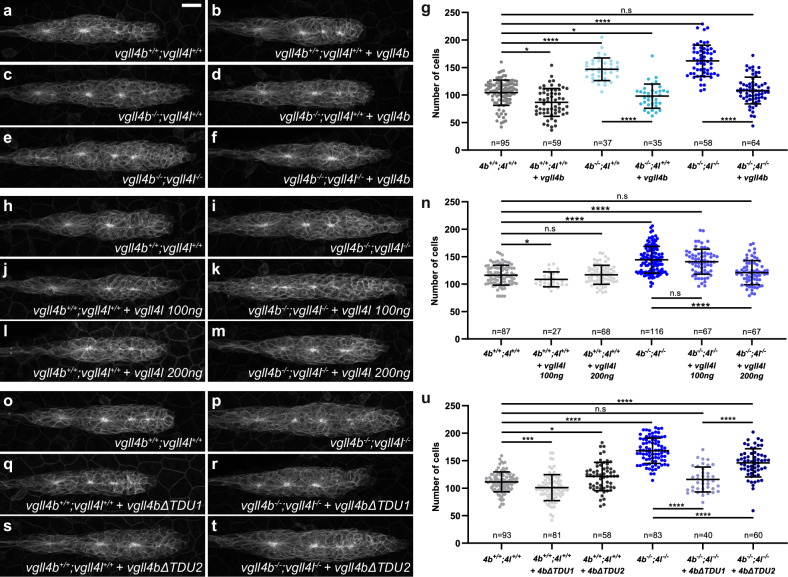
Vgll4b and Vgll4l are sufficient to rescue the loss of Vgll4 activity in the pLLP, and Vgll4b TDU2 is required for this function. MIP of confocal Z-stacks showing the pLLP in *vgll4b*^*+/+*^*;vgll4l*^*+/+*^ (**a**), *vgll4b*^*−/−*^*;vgll4l*^*+/+*^ (**c**), *vgll4b*^*−/−*^*;vgll4l*^*−/−*^ (**e**) embryos and the same genotypes injected with *tRFP-vgll4b* mRNA (**b**, **d**, **f**). **g** Quantification of pLLP cell number in each condition (*N* ≥ 2 independent replicates). MIP of confocal Z-stacks showing the pLLP in *vgll4b*^*+/+*^*;vgll4l*^*+/+*^ (**h**), and *vgll4b*^*−/−*^*;vgll4l*^*−/−*^ (**i**) embryos, injected with *vgll4l* mRNA at 100 ng/ μL (**j**, **k**) or at 200 ng/μL (**l**, **m**). **n** Quantification of pLLP cell number in each condition (*N* ≥ 2 independent replicates). MIP of confocal Z-stacks showing the pLLP in *vgll4b*^*+/+*^*;vgll4l*^*+/+*^ (**o**), and *vgll4b*^*−/−*^*;vgll4l*^*−/−*^ (**p**) embryos, injected with *tRFP-vgll4bΔTDU1* (**q**, **r**) or *tRFP-vgll4bΔTDU2* (**s**, **t**) mRNA. **u** Quantification of pLLP cell number in each condition (*N* ≥ 3 independent replicates). Data are presented as mean ± SD. Unpaired *t*-tests (Mann–Whitney) were conducted. Scale bar: 20 μm (**a**–**f**, **h**–**m**, **o**–**t**).

To further examine Vgll4 function in limiting pLLP cell number, we performed overexpression and rescue experiments. As expected, tRFP-Vgll4b fusion proteins localized to the nucleus (Supplementary Fig. 4c’). Overexpression of *vgll4b* mRNA in WT embryos resulted in a 20% reduction in pLLP cell number, indicating that Vgll4b is not only required but also sufficient to limit cell numbers in the pLLP (Fig. 4a, b, g). Furthermore, injection of WT *vgll4b* mRNA in *vgll4b*^*−/−*^ single mutants or *vgll4b*^*−/−*^;*vgll4l*^*−/−*^ double mutant embryos fully rescued the increase in pLLP cell number to control levels (Fig. 4a, c–g). Quantification of pLLP volume confirmed these observations (Supplementary Fig. 4a).

In contrast, injection of *vgll4l* mRNA at the same effective concentration as *vgll4b* had no effect on the pLLP cell number in WT embryos (Fig. 4h, j, n) and failed to rescue the increased cell count in *vgll4b*^*−/−*^*; vgll4l*^*−/−*^ mutants (Fig. 4i, k, n). Doubling the concentration of *vgll4l* mRNA fully rescued the double mutant phenotype (Fig. 4i, m, n) without affecting the pLLP cell number in WT embryos (Fig. 4h, l, n). These results indicate that while both Vgll4b and Vgll4l can restore Vgll4 function in the pLLP, Vgll4b exhibits greater potency. Consistent with this, only Vgll4b was sufficient to restrict pLLP cell number.

### TDU domains are differentially required for the tumor suppressor activity of Vgll4b

VGLL4 is the only VGLL family member containing two Tondu (TDU) motifs. The TDU motifs, particularly TDU2, have been shown to be necessary and, at least in some contexts, sufficient for the interaction of VGLL4 with TEAD, and its growth-inhibitory function^19,21,25^. To test whether Vgll4b’s growth-limiting activity in the pLLP depends on its interaction with Tead proteins, we generated *vgll4b* constructs lacking either the first or the second TDU domain (Supplementary Fig. 4f–h). Both variants localized to the nucleus, indicating that neither TDU motif is essential for nuclear localization (Supplementary Fig. 4d', e’). Injecting *vgll4bΔTDU1* mRNA in WT similarly resulted in a reduction in cell number as WT *vgll4b* overexpression (approximately 10%) and fully rescued *vgll4b*^*−/−*^;*vgll4l*^*−/−*^ double mutant phenotype (Fig. 4o–r, u). In contrast, injecting *vgll4bΔTDU2* in WT embryos led to a slight increase in cell count (approximately 9%) and only partially rescued the *vgll4b*^*−/−*^;*vgll4l*^*−/−*^ double mutant phenotype (Fig. 4o, p, s–u). These results indicate that, like Yap1, Vgll4b’s growth-limiting activity in the pLLP is Tead-dependent and that the functional Vgll4b-Tead interaction is largely mediated by TDU2.

### Vgll4b/l limits pLLP cell proliferation through Yap1 competition and Tead-mediated repression

**Fig. 5:**
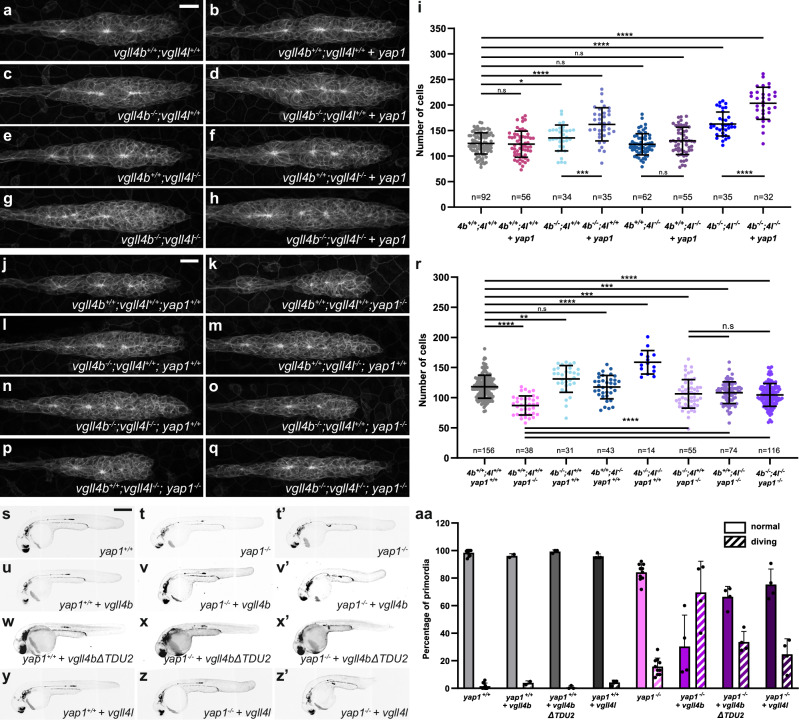
Vgll4 limits cell number in the pLLP through Yap1 Competition and Tead-Mediated Repression. MIP of confocal Z-stacks showing the pLLP in *vgll4b*^*+/+*^*;vgll4l*^*+/+*^ (**a**), *vgll4b*^*−/−*^*;vgll4l*^*+/+*^ (**c**), *vgll4b*^*+/+*^*;vgll4l*^*−/−*^ (**e**), *vgll4b*^*−/−*^*;vgll4l*^*−/−*^ (**g**) embryos and the same genotypes, respectively, injected with *tBFP-yap1* mRNA (**b**, **d**, **f**, **h**). **i** Quantification of pLLP cell number in each condition (*N* ≥ 2 independent replicates). MIP of confocal Z-stacks showing the pLLP in *vgll4b*^*+/+*^*;vgll4l*^*+/+*^*;yap1*^*+/+*^ (**j**), *vgll4b*^*+/+*^*;vgll4l*^*+/+*^*;yap1*^−/−^ (**k**), *vgll4b*^*−/−*^*;vgll4l*^*+/+*^*;yap1*^*+/+*^ (**l**), *vgll4b*^*+/+*^*;vgll4l*^*−/−*^*;yap1*^*+/+*^ (**m**); *vgll4b*^*−/−*^*;vgll4l*^*−/−*^*;yap1*^*+/+*^ (**n**), *vgll4b*^*−/−*^*;vgll4l*^*+/+*^*;yap1*^*−/−*^ (**o**), *vgll4b*^*+/+*^*;vgll4l*^*−/−*^*;yap1*^*−/−*^ (**p**), *vgll4b*^*−/−*^*;vgll4l*^*−/−*^*;yap1*^*−/−*^ (**q**) embryos. **r** Quantification of pLLP cell number in each condition (*N* ≥ 3 independent replicates, except for *vgll4b*^*−/−*^*;vgll4l*^*−/−*^*;yap1*^*+/+*^, *N* = 1). MIP of confocal Z-stacks showing overview images of 32 hpf *yap1*^*+/+*^ (**s**), *yap1*^*−/−*^ (**t**, **t’**), and embryos of the same genotypes injected with *tBFP-vgll4b* mRNA (**u**–**v’**), *tBFP-vgll4bΔTDU2* mRNA (**w**–**x’**), or with *vgll4l* mRNA (**y**–**z’**). **aa** Percentage of pLLP displaying normal migration or “pLLP diving” in each condition (*N* ≥ 3 independent replicates, except for *yap1*^*+/+*^ *+ vgll4b*, *N* = 2). Data are presented as mean ± SD. Unpaired *t*-tests (Mann–Whitney) were conducted. Scale bars: 20 μm (**a**–**h**, **j**–**q**) and 400 μm (**s**–**z’**).

Our previous results indicate that Yap1 and Vgll4b regulate pLLP growth through Tead-dependent mechanisms, prompting us to test whether they compete for Tead binding, as reported in other systems^19,24,25^. If true, Yap1 oncogenic activity should be enhanced in embryos lacking Vgll4. To test this, we overexpressed *yap1* in *vgll4* mutants. As shown before (Fig. 2g), overexpressing *yap1* in control *vgll4b*^*+/+*^*;vgll4l*^*+/+*^ embryos did not increase pLLP cell number (Fig. 5a, b, i). In contrast, *yap1* mRNA injection in *vgll4b*^*−/−*^ single or in *vgll4b*^*−/−*^;*vgll4l*^*−/−*^ double mutants further increased pLLP cell counts by 20 and 25%, respectively, compared to non-injected mutants (Fig. 5c, d, g–i). *yap1* overexpression in *vgll4l*^*−/−*^ single mutants did not lead to an increase in the cell number (Fig. 5e, f, i). This result indicates that Yap1 is more potent in the absence of Vgll4, indicating that Vgll4 competes with Yap1 for binding to Tead, thus limiting the proliferation-promoting function of Yap1 in the pLLP. It also confirms the asymmetric functional redundancy between *vgll4b* and *vgll4l*, with Vgll4b playing the dominant role in limiting Yap1 activity.

To further investigate the competition between Vgll4 and Yap1, we generated *vgll4b;yap1, vgll4l;yap1* double and *vgll4b;vgll4l;yap1* triple mutants to perform epistasis analyses. In line with the previously described Sd/Tead-mediated default repression by Tgi/Vgll4^23,26,27^, we anticipated that loss of Vgll4 would largely rescue the *yap1* mutant phenotype. Notably, loss of Vgll4b/l function only partially rescued pLLP cell numbers with *vgll4b*^*−/−*^*;yap1*^*−/−*^*, vgll4l*^*−/−*^*;yap1*^*−/−*^, and *vgll4b*^*−/−*^*;vgll4l*^*−/−*^*;yap1*^*−/−*^ showing approximately 30% more cells than their *yap1*^*−/−*^ siblings and 10% less than the WT control siblings (Fig. 5j–r). Thus, while loss of Vgll4 partially alleviates the growth defect caused by Yap1 depletion, consistent with a default repressor function, its inability to fully rescue the phenotype indicates that Vgll4b and Vgll4l also act as physical competitors that limit Yap1–Tead association and transcriptional output^23,26^.

To confirm that Vgll4b and Vgll4l are not acting solely by hindering Yap1-Tead physical interaction, we overexpressed *vgll4b* and *vgll4l* in the absence of Yap1 to test for further decrease in cell number. Injection of *vgll4b* in *yap1*^*−/−*^ mutant embryos led to a strong, early morphological phenotype, with many crippled embryos compared to the injection in their WT siblings (Fig. 5s–v’). While this precluded pLLP cell number quantification, it clearly showed that Vgll4b is more potent in the absence of Yap1 protein, confirming that Vgll4b functions beyond simply blocking Yap1-Tead interaction. In the malformed embryos, many pLLP deviated from their migration path, a low-penetrance phenotype we observed previously in *yap1* mutants and that we described as “diving” pLLP. While 1.29% of the WT embryos and 16% of the *yap1*^*−/−*^ mutant embryos showed “diving” primordia, this increased to 68% in *yap1*^*−/−*^ mutants injected with *vgll4b* mRNA (Fig. 5aa). *vgll4bΔTDU2* overexpression in *yap1*^*−/−*^ mutants resulted in significantly fewer “diving” primordia than *vgll4b*, indicating that this early phenotype does not require the presence of Yap1 but depends on the interaction of Vgll4b with Tead (Fig. 5w–x’, aa). Overexpression of *vgll4l* in *yap1*^*−/−*^ mutants caused a similar, but weaker effect, with 25% of the embryos showing “diving” primordia (Fig. 5y–z’, aa). Together, these results support a dual role for Vgll4b and Vgll4l as Tead-dependent default repressors and as physical competitors of Yap1, restraining Yap1-Tead-mediated transcriptional activity.

### Yap1 is active in the pLLP before migration

**Fig. 6:**
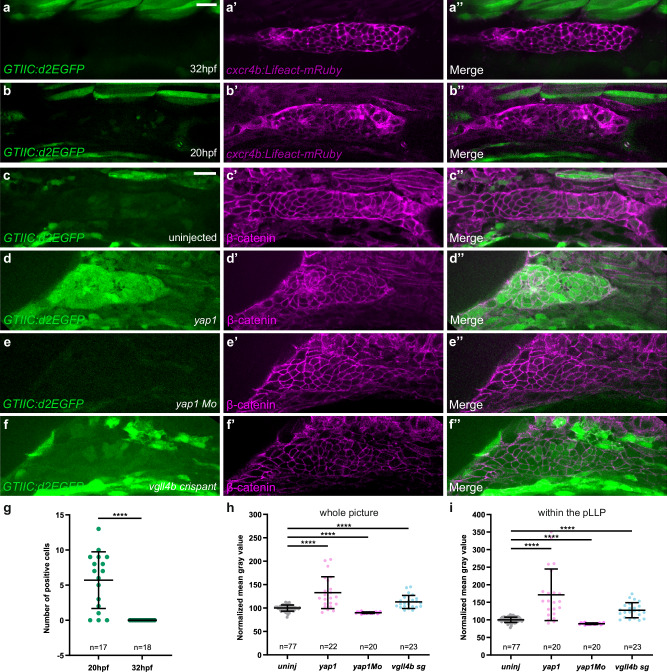
Yap1 transcriptional activity is detected in the pLLP prior to the onset of migration. **a**–**b”** Single-plane confocal images of the pLLP in control embryos at 32 hpf (**a**) and at 20 hpf (**b**) showing *GTIIC:d2EGFP* expression. *cxcr4b:Lifeact-mRuby* is used to visualize the pLLP (**a’**, **b’**). Merged images of the two channels are shown in (**a”**, **b”**). Single-plane confocal images of the pLLP in 20 hpf embryos (**c**), injected with *tBFP-yap1* mRNA (**d**), *yap1* Mo (**e**), or *vgll4b sg1*+*2* (**f**), showing *GTIIC:d2EGFP* (**c**–**f**), *β-catenin* (**c’**–**f’**), and merged channels (**c”**-**f”**). *β-catenin* is used to visualize the pLLP. **g** Quantification of *GTIIC:d2EGFP* positive cells in the pLLP of 20 hpf and 32 hpf control embryos (*N* = 1 independent replicate). Mean gray value measured either on the whole image (**h**) or within the pLLP on a single plane (**i**) in the GFP channel in the same embryos for each condition (*N* ≥ 1 independent replicates). Data are presented as mean ± SD. Unpaired *t*-tests (Mann–Whitney) were conducted. Scale bar: 20 μm (**a**–**f”**).

Given that Yap1 looked mostly cytoplasmic, and thus not transcriptionally active in pLLP cells during migration (Fig. 1h–l), we wanted to determine when Yap1 and Vgll4 are active in the lateral line system. Using the Tead activity reporter line *Tg(4xGTIIC:d2EGFP)*^*mw50*^ (*GTIIC:d2EGFP*, ref. ^48^), we found that the reporter activity was indeed very low or undetectable in the migrating pLLP of WT embryos (Fig. 6a–a”). However, weak and mosaic expression of the reporter was observed in the pLLP at 20 hpf prior to migration (Fig. 6b–b”). Quantification of the number of *GTIIC:d2EGFP* positive cells—defined as cells exhibiting significantly higher GFP fluorescence than the surrounding non-expressing tissue—confirmed that Yap1 activity was stronger in the pLL before or at migration onset (Fig. 6g). While *yap1* overexpression increased reporter activity by 70%, in the entire embryo and within the pLLP, *yap1* knock-down decreased it by 12%, confirming the specificity of the reporter for Yap1-Tead transcriptional activity (Fig. 6c–e”, h, i, Supplementary Fig. 5e, f). We then tested how a decrease in Vgll4b function would affect the reporter activity using CRISPR-Cas9-mediated knockdown with two guide RNAs (gRNA) targeting *vgll4b*. Both *vgll4b* gRNAs exhibited high efficiency as assessed by High-Resolution Melting analysis (HRM) (Supplementary Fig. 5a–d). Co-injection of *vgll4b* gRNAs significantly increased the *GTIIC:d2EGFP* fluorescence in the whole embryo and in the pLLP (27%), showing that *vgll4b* knockdown promotes Yap1-Tead transcription activity (Fig. 6c–c”, f–f”, h, i, Supplementary Fig. 5e, f). Altogether, these results indicate that Yap1 is transcriptionally active in the pLLP prior to migration and that this activity is limited by Vgll4b.

### Vgll4b interferes more than Vgll4l with Yap1-Tead transcriptional activity

**Fig. 7:**
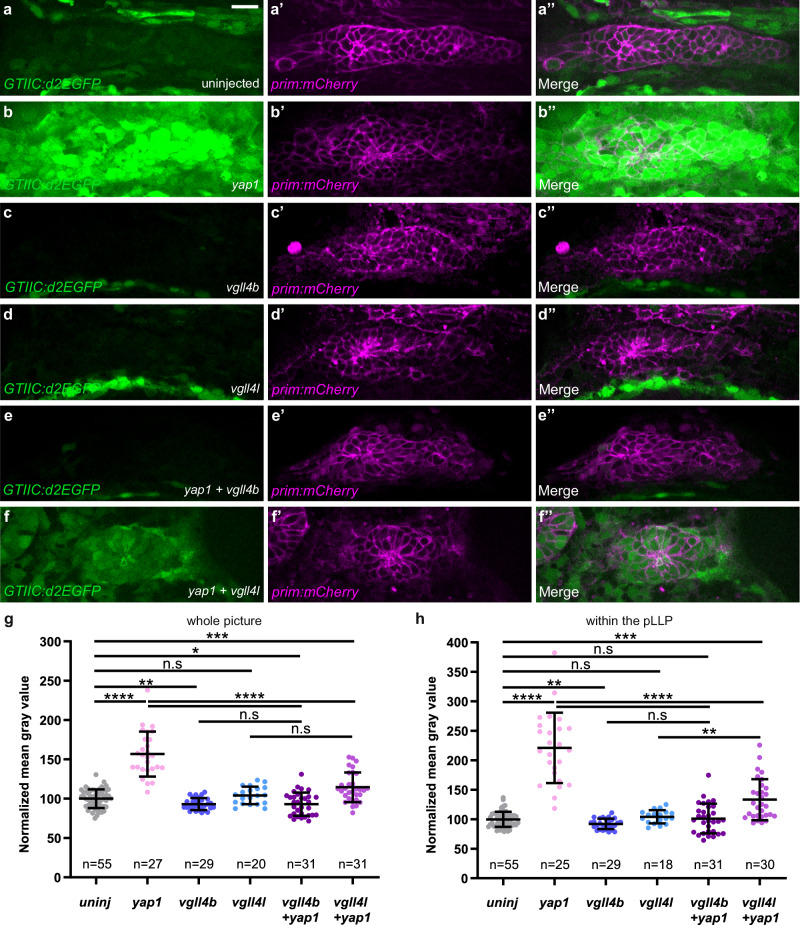
Vgll4b suppresses Yap1-Tead activity more efficiently than Vgll4l in the pLLP. **a**–**f”** Single planes confocal images showing the expression of *GTIIC:d2EGFP* in the pLLP of 20 hpf embryos, uninjected (**a**) or injected with *tBFP-yap1* (**b**), *tRFP-vgll4b* (**c**), *vgll4l* (**d**), *tBFP-yap1* and *tRFP-vgll4b* (**e**), *tBFP-yap1* and *vgll4l* (**f**) mRNA. The *prim-mCherry* transgene is used to visualize the pLLP (**a’**–**f’**). Merged images of the two channels are shown in (**a”**–**f”**). Quantifications of the mean intensity of *GTIIC:d2EGFP* measured on the average Z-stack projection of the whole image (**g**) or on a single plane within the pLLP in the same embryos (**h**) (*N* ≥ 1 independent replicates). Data are presented as mean ± SD. Unpaired *t*-tests (Mann–Whitney) were conducted. Scale bar: 20 μm (**a**–**f”**).

To further explore the interplay between the Yap1 and Vgll4 proteins, and potential functional differences between Vgll4b and Vgll4l paralogs, we performed overexpression experiments using the *GTIIC:d2EGFP* reporter line. As shown previously, *yap1* overexpression significantly increased the reporter activity (Fig. 7a–b”, g, h). In contrast, *vgll4b* overexpression resulted in a slight reduction of the *GTIIC:d2EGFP* signal by approximately 10% (Fig. 7c–c”, g, h, Supplementary Fig. 6a, b), while injection of *vgll4l* RNA at the same effective concentration had no noticeable effect (Fig. 7d–d”, g, h, Supplementary Fig. 6a, b). Notably, co-injection of *vgll4b* with *yap1* significantly reduced the *GTIIC:d2EGFP* signal compared to injection of *yap1* alone (compare Fig. 7e to 7b, Fig. 7g, h). Similarly, co-injection of *vgll4l* with *yap1* also reduced *GTIIC:d2EGFP* signal relative to *yap1* alone, though to a lesser extent than *vgll4b* co-injection (compare Fig. 7f to 7b and 7e, Fig. 7g, h). Taken together, these findings show that Vgll4b effectively outcompetes Yap1 and fully inhibits the strong increase in transcription resulting from *yap1* overexpression. Furthermore, these results confirm that Vgll4l functions in a similar manner, albeit with lower potency than Vgll4b.

### Yap1 and Vgll4b/l are required for proper formation of the pLL placode

**Fig. 8:**
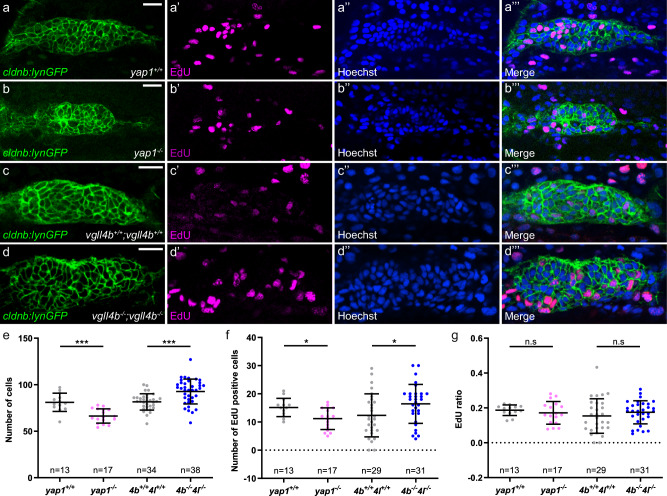
*yap1* and *vgll4b/l* are required for proper formation of the pLLP. Single plane confocal images showing the amount of EdU-stained nuclei in comparison to *cldnb:lynGFP* signal in *yap1*^*+/+*^ (**a**–**a”’**), *yap1*^*−/−*^ (**b**–**b”’**), *vgll4b*^*+/+*^*;vgll4l*^*+/+*^ (**c**–**c”’**), and *vgll4b*^*−/−*^*;vgll4l*^*−/−*^ (**d**–**d”’**) embryos at 19 hpf. Quantification of pLLP cell number (**e**), EdU positive cell number (**f**), and ratio of EdU positive cell number to total pLLP cell number (**g**) in each condition (*N* = 1 independent replicate for *yap1*, *N* =  3 independent replicates for *vgll4*). Data are presented as mean ± SD. Unpaired *t*-tests (Mann–Whitney) were conducted. Scale bar: 20 μm (**a**–**d”’**).

Our findings suggest that Yap1 and Vgll4 are required early in pLL formation to control the size of the pLLP. Since the pLL placode gives rise not only to the pLLP that delaminates from it, but also to the ganglion, differences in pLLP cell count might result from a cell specification defect in the placode. We thus quantified the number of cells in the pLL ganglion and pLLP in *yap1* and *vgll4b;vgll4l* mutants at 26 hpf. Despite a clear phenotype in the pLLP, ganglion cell numbers remained unchanged in both cases (Supplementary Fig. 7h–m), indicating that the pLLP cell count phenotypes in *yap1* (less cells) and *vgll4b;vgll4l* (more cells) mutants do not result from bigger, respectively smaller, ganglions. This result further suggests that the phenotype is already present at the onset of pLLP migration. Indeed, the pLLP in *yap1* mutant had 20% fewer cells than their control siblings at 19 hpf (20ss) (Fig. 8a–b”’, e). Furthermore, EdU assays indicated that proliferation rate was unchanged in *yap1* mutant embryos at that stage (Fig. 8f, g). Conversely, the pLLP of *vgll4b*^*−/−*^;*vgll4l*^*−/−*^ double mutants were 14% larger than their control siblings at 19 hpf (20ss) (Fig. 8c–e), but the proliferation rate also showed no significant difference (Fig. 8f, g). Altogether, these results indicate that Yap1 and Vgll4 are required in the pLLP prior to migration to regulate its number of cells and its size.

Surprisingly, while significantly smaller, *yap1* mutant pLLP exhibited significantly higher proliferation rates than their siblings during migration (Supplementary Fig. 7a–b”’, e–g). This indicates that increased proliferation during migration partially compensates for reduced cell count in delaminating pLLP, enabling complete migration and normal neuromast deposition^36^. We did not detect any significant changes in cell proliferation in *vgll4* double mutants at 32 hpf (Supplementary Fig. 7c–g).

### Changes in pLL placode size in *yap1* and *vgll4* mutants emerge between 15ss and 20ss

**Fig. 9:**
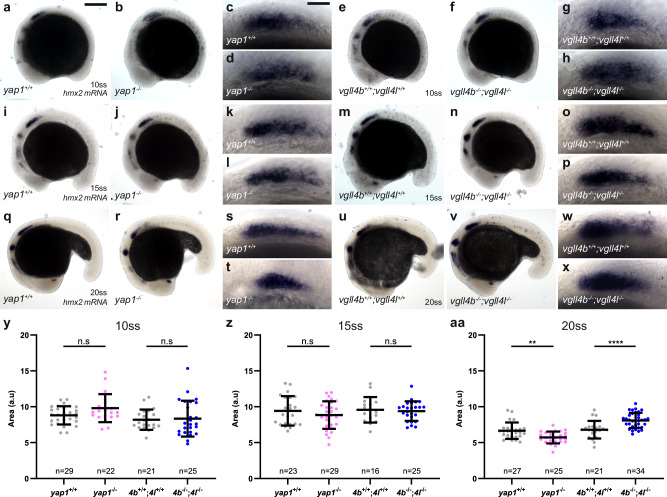
pLLP size changes in *yap1* and *vgll4* mutants emerge between 15ss and 20ss. Overview (**a**, **b**, **e**, **f**, **i**, **j**, **m**, **n**, **q**, **r**, **u**, **v**) and higher magnification images (**c**, **d**, **g**, **h**, **k**, **l,** **o**, **p**, **s**, **t**, **w**, **x**) taken on a brightfield microscope of *yap1*^*+/+*^ (**a**, **c**, **i**, **k**, **q**, **s**), *yap1*^*−/−*^ (**b**, **d**, **j**, **l**, **r**, **t**), *vgll4b*^*+/+*^*;vgll4l*^*+/+*^ (**e**, **g**, **m**, **o**, **u**, **w**), and *vgll4b*^*−/−*^*;vgll4l*^*−/−*^ (**f**, **h**, **n**, **p**, **v**, **x**) embryos stained by ISH for *hmx2* at 10ss (14 hpf) (**a**–**h**), 15ss (16.5 hpf) (**i**–**p**), and 20ss (19 hpf) (**q**–**x**). Quantifications of *hmx2* stained area in each indicated group at 10ss (**y**), 15ss (**z**), and 20ss (**aa**) (*N* = 1 independent replicate). Data are presented as mean ± SD. Unpaired *t*-tests (Mann–Whitney) were conducted. Scale bar: 400 μm (**a**, **b**, **e**, **f**, **i**, **j**, **m**, **n**, **q**, **r**, **u**, **v**), 20 μm (**c**, **d**, **g**, **h**, **k**, **l**, **o**, **p**, **s**, **t**, **w**, **x**).

To determine when Yap1 and Vgll4 are required to determine the size of the pLLP, we looked for reliable markers expressed in the pLL placode early in development. *hmx2* and *hmx3a* code for transcription factors essential for pLLP development and are expressed in the otic and lateral line placodes from 10-somites stage (ss) (14 hpf) onward^49–51^. Therefore, we used *hmx2* and *hmx3a* expression to quantify the size of the pLL placode, respectively pLLP, at 10ss, 15ss, 20ss, and 32 hpf in *yap1* and *vgll4* double mutants. At 10ss (14 hpf), no significant differences were observed in either mutant using either probe (Fig. 9a–h, y; Supplementary Fig. 8a–h, y). At 15ss (16.5 hpf), we observed no change with *hmx2*, but a trend toward reduced area in *yap1* mutants with *hmx3a* (Fig. 9i–p, z; Supplementary Fig. 8i–p, z). At 20ss (19 hpf), *yap1* mutants showed a smaller stained area, while *vgll4* mutants displayed a larger area with both probes (Fig. 9q–x, aa; Supplementary Fig. 8q–x, aa), consistent with our cell counts at this stage. These differences persisted at 32 hpf, validating *hmx2* and *hmx3a* as reliable markers to quantify the size of the pLLP (Supplementary Fig. 8ab–as). Together, these findings indicate that the phenotype emerges between 15ss (16.5 hpf) and 20ss (19 hpf).

### Yap1 activity is required between 10ss and 20ss for proper pLLP formation

**Fig. 10:**
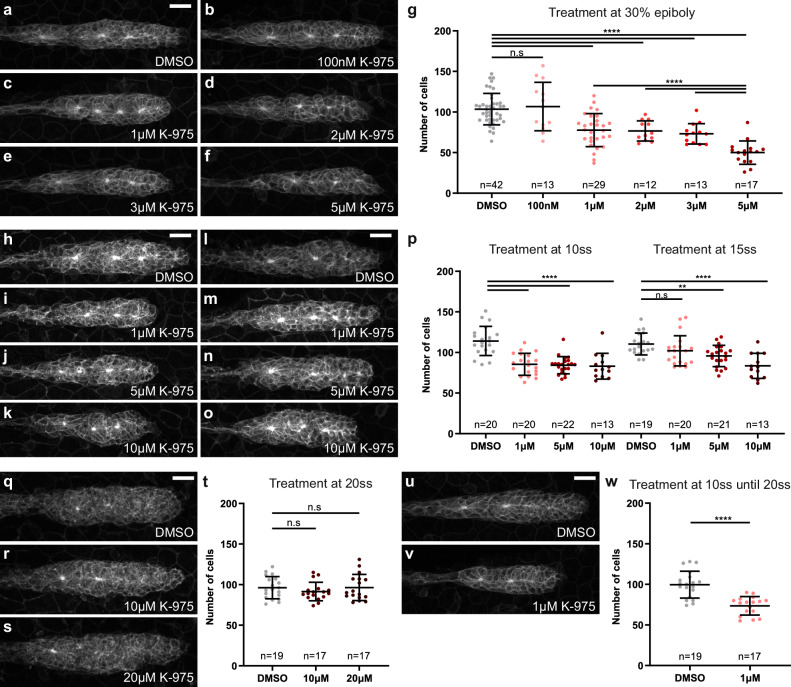
Yap1 activity is required between 10ss and 20ss for proper pLLP formation. MIP of confocal Z-stacks showing the pLLP in 32 hpf embryos treated at 30% epiboly with DMSO (**a**) or increasing concentrations of K-975: 100 nM (**b**), 1 μM (**c**), 2 μM (**d**), 3 μM (**e**), and 5 μM (**f**). **g** Quantification of pLLP cell number at 32 hpf in the indicated groups (*N* = 1 independent replicate, except for DMSO and 1 μM K-975, *N* = 3). MIP of confocal Z-stacks showing the pLLP of 29 hpf embryos treated at 10ss (14 hpf) (**h**–**k**) or at 15ss (16.5 hpf) (**l**–**o**) with DMSO (**h**, **l**) or 1 μM (**i**, **m**), 5 μM (**j**, **n**), and 10 μM (**k**, **o**) K-975. **p** Quantification of pLLP cell number at 29 hpf in the indicated groups (*N* = 1 independent replicate). MIP of confocal Z-stacks showing the pLLP of 29 hpf embryos treated at 20ss (19 hpf) with DMSO (**q**) or 10 μM (**r**) and 20 μM (**s**) K-975. **t** Quantification of pLLP cell number at 29 hpf in the indicated groups (*N* = 1 independent replicate). MIP of confocal Z-stacks showing the pLLP of 29 hpf embryos treated from 10ss (14 hpf) to 20ss (19 hpf) with DMSO (**u**) or 1 μM K-975 (**v**). **w** Quantification of pLLP cell number at 29 hpf in the indicated groups (*N* = 1 independent replicate). Data are presented as mean ± SD. Unpaired *t*-tests (Mann–Whitney) were conducted. Scale bar: 20 μm (**a**-**f**, **h**-**o**, **q**-**s**, **u**, **v**).

To confirm this early requirement of Yap1 and precisely define the developmental window during which it is required to control the size of the pLLP, we performed pharmacological treatments, using K-975, a small molecule that specifically inhibits YAP1/WWTR1 interaction with TEAD^52–54^. We first treated embryos between 30% epiboly and 32 hpf, before imaging. K-975 treatment led to a significant reduction in pLLP cell counts, resembling the phenotype observed in *yap1* mutants. This reduction was dose-dependent (Fig. 10a–g). To assess K-975 specificity in our model, we evaluated its effect on the *GTIIC:d2EGFP* reporter line. Embryos were treated at 30% epiboly and imaged at 20 hpf. The reporter signal intensity was significantly reduced throughout the embryo and within the pLLP following K-975 treatment (Supplementary Fig. 9a–d). Next, we performed temporally controlled drug treatments between 10ss (14 hpf) and 20ss (19 hpf). WT embryos were treated from the desired stage until imaging at 30 hpf. When treated at 10ss (14 hpf), we observed a strong reduction in pLLP cell counts, with 1, 5, and 10 µM K-975 (Fig. 10h–k, p). Interestingly, when treated at 15ss (16.5 hpf), 1 µM K-975 no longer affected cell number. Treatment with 5 µM K-975 led to a mild reduction, and 10 µM produced an effect comparable to the 10ss (14 hpf) treatment, suggesting that Yap1 activity begins to decline between 10ss (14 hpf) and 15ss (16.5 hpf) (Fig. 10l–p). This was further confirmed by treating embryos from 20ss (19 hpf) onward, where we observed that neither 10 µM nor 20 µM K-975 led to any measurable effect on pLLP cell number (Fig. 10q–t). Finally, 1 µM K-975 treatment limited to the 10ss–20ss (14–19 hpf) window was sufficient to reduce pLLP cell number to the same extent as continuous treatment until imaging, confirming that Yap1 activity is required during this early time window (Fig. 10u–w).

## Discussion

In this study, we uncovered that the number of cells and size of the pLLP are precisely regulated by a balance between Yap1 growth-promoting and Vgll4 growth-limiting activities. Yap1, in association with Tead proteins, ensures that the pLLP contains a sufficient number of cells before migration begins. Conversely, Vgll4b and Vgll4l partially redundantly limit the number of cells in the pLLP, with Vgll4b exhibiting stronger tumor-suppressor activity. Our results suggest that Vgll4 limits pLLP size in two ways: as an allosteric competitor that inhibits the transcriptional activity of the Yap1-Tead complex and as an active repressor with Tead proteins. Overall, our findings bring together the two previously proposed models for VGLL4 activity, namely that of a physical competitor for YAP1/TEAD-mediated transcription and a default repressor^19,21,23,25,26,55^, demonstrating that both modes of action can operate simultaneously to regulate normal organ growth in vivo. Our results provide an integrated framework explaining how VGLL4 fine-tunes TEAD transcriptional output and ensures robust control of tissue growth.

During embryonic development, spatio-temporally coordinated cell proliferation is necessary for well-proportioned organ formation and growth. We previously showed that the Hippo signaling pathway effector Yap1 is required for appropriate pLLP cell number^36^. Here, we show that without Yap1 activity, the pLLP is already smaller prior to migration. By combining pharmacological treatment at specific times with analysis of mutants at early stages, we show that Yap1 activity is required as early as the 10ss (14 hpf), but not after the 20ss (19 hpf), revealing a specific and temporally restricted requirement for Yap1 activity prior to pLLP migration. Previous work has shown that pLL precursors undergo a generalized proliferation phase during gastrulation (before 10 hpf), followed by a transient quiescent period until about 16 hpf, when a second, more restricted wave of proliferation occurs within the forming placode^56^. Importantly, only the pLLP lineage experiences this later proliferative burst, as ganglion precursors become post-mitotic between 8 and 13 hpf^57^. This is also consistent with studies showing that retinoic acid (RA) signaling is required between 8 and 10 hpf to specify the correct pool of shared pLL precursors: RA inhibition during this early window reduces both neuromast and ganglion size, indicating that the two lineages are not yet separated and that their initial cell number is jointly controlled by RA-dependent regulation of the first proliferation phase^30,56^. Our finding that Yap1 is required later—between the 10ss and 20ss (14–19 hpf)—and that ganglion size is unaffected in *yap1* and *vgll4b/l* mutants (Figs. 9, 10 and Supplementary Figs. 7h–m, 8), suggests that Yap1 acts specifically on the pLLP lineage slightly before or during the second proliferation phase. Thus, Hippo pathway activity does not participate in the early RA-dependent expansion of the shared precursor pool, but instead refines pLLP size during a later, lineage-restricted proliferative window. Once the initial pLLP cell number has been established before migration, Wnt/β-catenin signaling maintains proliferating progenitors in the pLLP leading region during migration^31,32^. This partially compensates for cell loss due to neuromast deposition, ensuring that the pLLP can deposit a complete set of lateral line organs^34,35^. Here, we show that in *yap1* mutants, proliferation is increased during migration. This partially compensates for the reduced cell number in the delaminating pLLP, enabling the pLLP to deposit neuromasts and finish migration (Fig. 8 and Supplementary Fig. 7a–g). Further investigation will be required to uncover the compensating mechanism.

YAP1 is a transcriptional cofactor requiring DNA-binding proteins to activate gene expression. Although transcription factors of the TEAD family are the main DNA-binding partners, YAP1 can interact with other transcription factors, such as the RUNX2, p73, AP-1, β-CAT^58–60^. Here, we show that the proliferation-promoting function of Yap1 in the lateral line system is fully Tead-dependent, since (i) the Tead-binding defective *yap1*^*bns22*^ allele shows the same reduction in cell count as the null mutant allele and (ii) a Tead-binding defective Yap1 cannot restore the cell number when injected in *yap1* mutants, while a WT Yap1 does. This Tead-dependency is also supported by quantifications of the *GTIIC:d2EGFP* reporter activity in the pLLP upon Yap1 overexpression and knockdown. While we cannot exclude contributions from adjacent tissue that show Yap1 nuclear localization and Yap1-Tead activity, these results support a primarily cell-autonomous requirement for Yap1 in the pLLP progenitors (Figs. 6 and 7). Tissue-specific manipulations would be needed to directly test potential non-cell-autonomous effects.

VGLL4 functions by competing with YAP1 to bind TEAD proteins, interacting with TEAD in the same region as YAP1. This competition inhibits YAP1-TEAD transcriptional complex formation dose-dependently, thereby suppressing the activation of genes involved in cell proliferation and survival^19,21,23,25,61,62^. Our findings align with the established role of VGLL4 in mammals, with Vgll4b and Vgll4l competing with Yap1 for Tead binding, thereby restricting Yap1’s growth-promoting activity in the pLL. First, we show that Vgll4b activity in the pLL depends on the TDU2 motif, which mediates the interaction of VGLL4 with TEAD^19,25^. Second, the increase in cell number in *vgll4* mutants is largely rescued by Yap1 loss (in double and triple mutants), indicating increased Yap1 activity upon loss of Vgll4 activity. Third, we show that Yap1 is more potent when overexpressed in *vgll4* mutants than in WT, again strongly suggesting that Vgll4 limits Yap1 activity. Altogether, our results indicate that Vgll4b and Vgll4l restrain pLLP growth by competing with Yap1 to limit its capacity to form an active transcriptional complex with Tead DNA-binding proteins. Which of the four Tead proteins are partners of Yap1, Vgll4b, and Vgll4l in the LL system remains unknown.

However, our results suggest a more complex role for Vgll4 in regulating cell number in the pLLP. Indeed, if Vgll4b and Vgll4l acted only by physically preventing Yap1-Tead interaction and inhibiting their transcriptional activity, then Vgll4 loss- or gain-of-function in *yap1* mutants should have no additional effect over the loss of *yap1* alone. Instead, we found that losing either *vgll4b* or *vgll4l*, or both, partially rescues the reduced cell counts in *yap1* mutant pLLP (Fig. 5j–r). These results align with the classical model of default repression, showing that VGLL4/Tgi primarily functions as a default repressor in physiological contexts, with YAP1/Yki competing with VGLL4/Tgi repression in a dose-dependent manner. Indeed, Drosophila *tgi* mutants^23^ and mice lacking VGLL4 function in the liver^26^ exhibit no overt phenotypes. However, in both cases, the loss of Tgi/VGLL4 rescues the Yki/YAP1 loss-of-function, demonstrating that the primary role of Yki/YAP1 in these contexts is to counteract Tgi/VGLL4-mediated default repression^23,26^. While, similarly, our data show that the loss of Vgll4 activity partially rescues the reduced cell number in *yap1* mutant pLLP, demonstrating that Vgll4b and Vgll4l function in part as default repressors, the loss of *vgll4b* and *vgll4b/l* activity on its own has a phenotype showing that Vgll4 function is required in the presence of transcriptionally active Yap1 to compete with it and limit its activity. Our data therefore support a dual role for Vgll4b/l in the pLLP, functioning simultaneously as default repressors and as competitors of Yap1-Tead-mediated transcription to regulate cell number and organ size.

Furthermore, overexpression of *vgll4b* in *yap1* mutants caused severe early morphological defects, absent when *vgll4b* was overexpressed in WT. These observations confirm that, in addition to counteracting Yap1 transcriptional activity, Vgll4 proteins can modulate Tead function directly, influencing gene expression even in the absence of Yap1. This is also in agreement with the significant increase in the number of “diving” pLLP we observed upon *vgll4b* overexpression in *yap1* mutants, a phenotype occurring at much lower penetrance in *yap1* mutants (Fig. 5s–aa). This phenotype may result from an enhanced endogenous Vgll4b repressive activity when Yap1 is unavailable to compete. This is consistent with the role of human VGLL4, being indispensable for repressive function of TEAD4 in adipogenesis independent of the presence of YAP1^55^. Furthermore, very recent data show that VGLL4 can induce the assembly of large TEAD4 condensates, inducing DNA aggregation and transcriptional repression^63^. Together, these results indicate that Vgll4b and Vgll4l limit cell number in the pLLP both by competing with Yap1-Tead transcription and by Tead-mediated repression. Moreover, our results highlight the importance of maintaining a precise balance between Yap1 and Vgll4 binding to Teads, likely modulated by factors such as their relative abundance and their binding affinities for Teads.

An alternative, non-exclusive hypothesis is that Vgll4 proteins compete with both Yap1 and another factor, potentially its homolog Wwtr1. Although Wwtr1 alone is not required for pLLP size control, its function may be required in the absence of Yap1, as it is expressed during pLLP migration (ref. ^36^ and Supplementary Fig. 1d, g–h). Yap1 and Wwtr1 function redundantly for posterior body axis morphogenesis and epidermal basal cells in zebrafish^39,64^. This could also apply to the pLLP, though testing it remains challenging due to the severe early morphological defects of the *yap1;wwtr1* double mutant embryos.

The zebrafish genome encodes three Vgll4 paralogs—*vgll4a*, *vgll4b*, and *vgll4l*. Previous studies have reported *vgll4b* and *vgll4l* expression in the pLL, suggesting their possible roles in regulating cell migration and/or proliferation. In particular, *vgll4b* is strongly and continuously expressed throughout migration and in deposited neuromasts^45^. Our findings show that Vgll4b and Vgll4l are required and function partially redundantly to limit cell numbers in the pLLP. Interestingly, Vgll4b plays a more predominant role than Vgll4l. Vgll4b alone is necessary and sufficient to limit the number of cells in the pLLP, not Vgll4l alone (Fig. 3g–m). Additionally, half the amount of *vgll4b* mRNA, compared with *vgll4l*, rescues the number of cells in the pLLP upon total loss of Vgll4 activity (Fig. 4h–n). This functional difference aligns with sequence homology analysis, indicating that Vgll4b is much closer to the human VGLL4 protein than Vgll4l (70 versus 32% identity, ref. ^45^). This higher degree of conservation suggests that Vgll4b could also have higher affinity for Tead and greater propensity to compete with Yap1 for binding to Tead. Our findings support this, showing that upon co-expression with Yap1, Vgll4b is more potent than Vgll4l in interfering with Yap1-Tead transcriptional activity measured by the *GTIIC:d2EGFP* reporter (Fig. 7). Further investigations into the molecular mechanisms and signaling pathways underlying the distinct functions of Vgll4b and Vgll4l will be crucial in elucidating their roles within the Yap1-Tead regulatory axis. Comparative studies examining binding kinetics, cofactor associations, and target gene regulation could provide valuable insights.

## Materials and methods

### Zebrafish husbandry and generation of mutant and transgenic lines

Procedures involving animals were carried out according to the guidelines of the Goethe University of Frankfurt and were approved by the German authorities (Veterinary Department of the Regional Board of Darmstadt). We have complied with all relevant ethical regulations for animal use. Sex was not considered in the study design because the experiments were conducted in early-stage zebrafish embryos, prior to sexual differentiation. Zebrafish were anesthetized using a 168 mg/L concentration of tricaine (MS-222, ethyl 3-aminobenzoate methanesulfonate) and euthanized using an overdose of tricaine (300 mg/L) or hypothermic shock, according to the German Animal Welfare Ordinance and the SOP of the animal facility at Goethe University’s Riedberg campus.

Embryos were staged as previously described^65^. *yap1*^*fu48*^^36^, *yap1*^*bns22*^^39^, and *wwtr1*^*fu55*^^66^ mutant lines and *Tg(-8.0cldnb:lynEGFP)*^*zf106*^ (*cldnb:gfp*)^67^, *Tg(4XGTIIC:d2EGFP)*^*mw50*^^40^, *Tg(prim:mem-mCherry)* (*prim:mCherry*)^68^ transgenic lines were characterized previously.

The *vgll4l*^*fu93*^ allele was generated using TALEN targeting exon 2 as previously described in refs. ^36,66^. The *vgll4b*^*bns23*^ allele was generated using CRISPR-Cas9 with the following sgRNA—5′-GCAGTCAGCAACCACCGGAC-3′ targeting exon 2 of the *vgll4b* gene. sgRNA DNA oligos were cloned into the pT7-gRNA vector (Addgene plasmid #46759) and in vitro transcribed with the MEGAshortscript T7 transcription kit (Ambion). *CAS9* mRNA was obtained by in vitro transcription of linearized *pT3TS-nCas9n* vector (Addgene plasmid #46757) with MEGAscript T3 transcription kit (Ambion). 100 pg of sgRNA and 150 pg of *CAS9* mRNA were co-injected into 1-cell stage AB embryos. The *Tg(cxcr4b(FOS):Lifeact-mRuby)*^*fu16*^ (*cxcr4b:lifeact-mRuby*) was generated using BAC homologous recombination by replacing the second exon of *cxcr4b* in the *cxcr4b*-containing fosmid CH1073-406F3 by *Lifeact-mRuby* followed by zebrafish transgenesis as described in refs. ^69,70^.

### Single embryo genotyping

Embryos were retrieved from mounting stamps, as described in ref. ^81^. Single fixed/live embryos were boiled at 95 °C in 50 mM NaOH (Stock 1 M in ddH_2_O) for 1 h and then neutralized by adding 1/10th of the volume of 1 M Tris-Cl (pH 8.2). After NaOH neutralization, 2 μl of this crude preparation was used for PCR genotyping^36,66^. Genotyping of *yap1*^*fu48*^ and *wwtr1*^*fu55*^ was performed as described previously^36,66^. Genotyping of *vgll4b*^*bns23*^, *vgll4l*^*fu93*^, and *yap1*^*bns22*^ was performed using primers listed in Table S1. The *vgll4l* and *yap1*^*bns22*^ PCR products did not require further digestion and were loaded directly onto 4% and 3% agarose gels, respectively. The *vgll4b* PCR product was digested with PstI overnight before loading onto a 2% gel.

### Morpholinos

Splice-blocking *yap1* Mo (5′-AGCAACATTAACAACTCACTTTAGG-3′) was injected at 0.5 mM with a drop size of 2.1 nL^36,71^.

### RNA extraction, cDNA synthesis, and RT-PCR

RNA extraction was performed using TRIzol reagent according to manufacturer’s instructions. Embryos at 32 hpf were dechorionated and homogenized in 500 μL TRIzol with a blunt needle tip syringe (20–40 embryos per tube). cDNA was generated using the iScript^TM^ cDNA Synthesis Kit (Bio-Rad, 1708890) for qPCR or the Invitrogen Superscript III or IV for RT-PCR according to manufacturer’s instructions. The cDNA was diluted 1:2 to obtain a final concentration of 25 ng/μl when used for primer efficiency assays or 1:4 to obtain a final concentration of 12.5 ng/μl when used for comparative gene expression analyses.

Primers were designed using Primer-BLAST and the online OligoAnalyzer tool from Integrated DNA Technologies (IDT) to identify the most optimal primer pairs^72^. Primer efficiency tests were performed to determine the ability of the primers to reliably and accurately bind to the correct sequence^73^. cDNA diluted for primer efficiency assays was further serially diluted 1:2 to obtain a total of six concentrations (25, 12.5, 6.25, 3.125, 1.5625, and 0.78125 ng/µL). For each primer pair, all concentrations were tested in triplicates. Real-time qPCR was performed using the Bio-Rad iTaq Universal SYBR Green Supermix (1725270) in a CFX Connect Real-Time System (Bio-Rad). Reaction mixes and programs were standardized for all primers, which are listed in Table S1. The primer sequences used for the qPCR experiments are listed in Table S1. Fold changes were calculated using the ΔΔCt method^74^. The expression of the genes of interest was normalized to that of Rpl13.

### Cloning full length and mutated forms of *yap1, vgll4b, vgll4l*, and ISH probes

All primers used for cloning are listed in Table S1. Full-length cDNAs encoding *yap1*, *vgll4b*, and *vgll4l*, and partial cDNA encoding *vgll4a*, *vgll4b*, *vgll4l, hmx2*, and *hmx3a* were amplified using 30 hpf whole embryo cDNA. PCR products were ligated into pCS2-derived vectors (for full-length cDNA) or in pGEM-T (Promega) according to the manufacturer’s instructions (for ISH probes). *yap1S54A*, *vgll4bΔTDU1*, and *vgll4bΔTDU2* were generated by overlapping PCR using the primers listed in Table S1. Capped mRNAs were transcribed using the Sp6 mMessage mMachine kit (Invitrogen, AM1340).

### Cas9 sgRNA guide design and microinjections

Gene-specific guide RNAs (sgRNA) were designed using the online tool CRISPOR^75^. Suitable sgRNAs were selected and synthesized as previously described^76,77^ DNA templates used to transcribe sgRNA were produced by annealing 1 µl of CRISPR-Cas9 constant oligo (AAAAGCACCGACTCGGTGCCACTTTTTCAAGTTGATAACGGACTAGC-CTTATTTTAACTTGCTATTTCTAGCTCTAAAAC) and 1 µl of a variable gene specific oligo (*vgll4b* – Exon2-1: TAATACGACTCACTATAGGCGAACAGGCGGCTCATCTGTTTTAGAGCTAGAA, *vgll4b* – Exon2-2: TAATACGACTCACTATAGGCAGTCAGCAACCACCGGACGTTTTAGAGCTAGAA) (10 μl total; 95 °C 5 min, 95–85 °C with −2 °C/s, 85–25 °C with 0.1 °C/s) followed by a fill-in reaction, 2.5 µl dNTPs (10 mM), 2 µl 10x NEB r2.1, 1 µl NEB T4 DNA Polymerase (10 μl total, 11 °C 20 min). The products were purified using the GeneJET PCR Purification Kit (Thermo Fisher Scientific). gRNA was transcribed using the MEGAscript T7 transcription Kit (Thermo Fisher Scientific) and purified using the RNA Clean & Concentrator Kit (Zymo Research). Microinjection was performed in 1-cell stage embryos with a total injected amount of 130 pg *Cas9 mRNA* (kind gift of Thomas Thumberger and Jochen Wittbrodt) and 260 pg of each sgRNA per embryo. Genomic DNA was extracted at the developmental stage of interest.

### High-resolution melting (HRM)

HRM was performed according to ref. ^78^. Primers were designed using the Primer3 tool (NCBI) to produce a product of 75–150 bp spanning the Cas9 targeting site (Table S1). All reactions were set up with 5 µl of iTaq Universal SYBR Green Supermix (Bio-Rad #1725120), 0.5 µl of primer pair (10 mM each), 2 µl of genomic DNA, and water up to 10 µl. PCR and HRM were performed in a CFX Connect Real-Time PCR Detection System (Bio-Rad) using 96-well plates using the following program: 95 °C for 7 min, then 35 cycles of 95 °C for 10 sec and 60 °C for 30 s (Two-step PCR), followed by an increase from 55 to 95 °C with a gradual increment of 0.1 °C/s (HRM). The analysis was performed using Precision Melt Analysis (Bio-Rad).

### Anti-Yap1 antibody production

To generate the antibody against zebrafish Yap1, the fragment of Yap1 encoding the first 230 amino acids was cloned into the expression vector *pGST-MCS-6XHIS*. The resulting *pGST-Yap1 (1-230)-6XHIS* was then produced in large quantities (BIOSS Toolbox Core Facility, Freiburg, Germany) and shipped to Eurogentec (Belgium) to immunize two rabbits pre-selected for their low pre-immune serum background. After the immunization program, different serum samples were received and directly tested by immunostaining on WT and *yap1* mutant embryos.

### Whole-mount immuno-fluorescence and whole-mount in situ hybridization (WISH)

For whole-mount immunostaining, embryos were fixed overnight at 4 °C or room temperature for 2 h in 4% PFA in 1X PBS (pH=7.4). After fixation, embryos were washed with PBS-T (PBS + 0.1% tween-20) for 5 × 5 min. Fixed embryos were used directly for the experiments without upgrading them in methanol. The rest of the procedure was performed according to standard procedures^79^. In this study, rabbit anti-Yap1 (1:200, VL7801), rabbit anti-Taz (1:200, Cell Signaling D24E4)^66^, mouse anti-βcatenin (1:200, Santa Cruz Biotechnology, 15B8), and mouse anti-GFP (1:500, Takara Bio Europe/France, JL8)^80^ were used as primary antibodies. Anti-rabbit and anti-mouse Alexa-conjugated antibodies were used as secondary antibodies (1:500, Molecular Probes, Eugene, OR, USA).

Whole-mount in situ hybridization was performed according to standard procedures^79^. Embryos at 10, 15, and 20ss (14, 16.5, and 19 hpf) were treated with pK for 20 min, whereas 32 hpf embryos were treated for 45 min. The *vgll4a*, *vgll4b vgll4l, hmx2* and *hmx3a* probes were used at 1:200 dilution. To visualize the pLLP after WISH, an immunostaining with the mouse anti-GFP antibody was performed as explained above.

### EdU (5-ethynyl-2′-deoxyuridine) treatment

The embryos were dechorionated and incubated with 10 mM EdU solution (Click-iT EdU, C10338, Life Technologies GmbH and Invitrogen) for 20 min on ice. After washing, embryos were incubated at 28.5 °C for 1 h to allow EdU incorporation and treated with the Click-iT reaction solution following the manufacturer’s instructions.

### Pharmacological treatments

A 10 mM stock solution of K-975 (HY-138565, MedChemExpress) was prepared in dimethylsulfoxide (DMSO). For embryo treatment, the stock solution was diluted in E3, and controls were treated with a corresponding concentration of DMSO in E3.

### Image acquisition

Imaging of live and fixed embryos was performed on a Nikon W1 Spinning Disc Microscope with the following objectives: 10x air objective (NA 0.45, WD 4 mm), 20x (NA 0.95, WD 0.95 mm), and 40x (NA 1.15, WD 0.60 mm) water objectives. The embryos were mounted as described in ref. ^81^, with the following modifications: no second layer of 0.5% LMPA was added on top of the 0.3% LMPA. The image recording software used was NIS-Elements v4.

Imaging of ISH embryos was performed under an inverted microscope (Ti-S) using a 4x objective to acquire whole embryos and a 20x objective for close-ups of the pLLP.

### Image processing and quantification of cell numbers in the pLLP

Unless otherwise indicated, all cell counts were performed when the pLLP reached the middle of the yolk extension in control and mutant embryos (approximately 32 hpf). Cell counts at an earlier developmental stage were performed on embryos between 18 and 22ss. All images were processed using the Fiji software.

For fixed samples and injections of full-length and mutated forms of *yap1* in the *yap1* mutants (Fig. 2, Supplementary Fig. 2), the number of cells in the pLLP was manually counted using the Cell Counter plugin.

For all other live acquisitions, automated cell counting was performed using the GFP signal of the *cldnb:gfp* transgenic embryos. Single cells of the pLLP were segmented in 3D using a homemade Fiji plugin based on MorphoLibJ and 3D manager built-in plugins. First, the plugin registers the pLLP in X and Y, and cropping in Z. This is performed by generating a maximum Z-projection, which is then blurred. The image is segmented in 2D using a minimum threshold and rotated by the angle formed by the difference between the long axis of the ellipsoid and the horizontal axis. After the rotation, the image is cropped. Once the pLLP is registered, single-cell segmentation starts on the Z-stack image using the MorphoLibJ plugin after a step of 3D Gaussian blur. Filtering and clearing are then performed to remove segments below or above a certain volume threshold and clear blank slices in Z. Finally, single-cell measurements are obtained using the 3D Manager plugin and saved in the output files. Manual checking of the quality of 3D segmentation was systematically performed. Inaccurate segmentations (under- or over-segmentation) were removed from the analysis.

R v3.6.1 was used to compile the segmented data. A homemade R script allowed for the removal of individual cells from the dataset, which were likely not part of the pLLP (wrongly segmented skin cells). The detection of these outliers was based on volume and surface/volume ratio. The cell count and total volume of the pLLP were extracted from these datasets.

### Neuromast deposition

Neuromast deposition pattern analysis was performed using a custom Fiji macro script that segments individual cell clusters and the pLLP. The images were segmented based on optimized filter parameters derived from trial and error. After segmentation, the macro allows for manual correction. The ROIs and their corresponding positions were saved in output files. R v3.6.1 was used to compile cell cluster data. Using a homemade R script, the positions of the neuromasts were normalized to the initial detected cell cluster (ganglion), and the number of neuromasts deposited was calculated. Additionally, the number of neuromasts deposited at the end of migration was manually counted on independent datasets.

### Rosette detector

The method used for rosette detection is based on a convolutional neural network (CNN)^82^ and was modified from the “rosette detector” algorithm previously used in the laboratory and described in refs. ^83,84^. The algorithm was updated with a state-of-the-art CNN using Caffe as a backend^82^ and re-trained with manually labeled primordia. The script was launched in Visual Studio Code (V1.53.2). Using maximum Z-projection images as an input, the algorithm detects structures similar to the rosettes and assigns them a score indicative of similarity to the WT training data. The number of detections and weighted (sum of detection scores) detections were saved in a .csv output file. R v3.6.1 was used to calculate the ratio of weighted detections over the number of detections to reflect the “rosettiness” of a pLLP.

### Aspect ratio measurement

Aspect ratio measurements were performed by manually encircling the pLLP on maximum Z-projection images using the freehand selection tool in Fiji. Shape descriptors were selected in the Set Measurements menu, which yielded measures of circularity, aspect ratio (AR), roundness, and solidity.

For all analyses, the processed data were extracted from R as .csv files and imported into GraphPad Prism for plotting.

### Statistics and reproducibility

Sample size was determined by checking the stability of the standard deviation. For Figs. 1–5 and Supplementary Figs. 1–4, we excluded embryos presenting inaccurate segmentations (under- or over-segmentation). Data were replicated at least 3 independent times when relevant. We did not use randomization. For Figs. 6–10 and Supplementary Figs. 5–9, area, intensity signal, and cell counts were performed blinded to group allocation and by several of the authors reaching the same conclusion.

GraphPad Prism was used for statistical analysis, and samples were subjected to the two-tailed unpaired t-test (also called Mann–Whitney test) to determine the *P* values. Statistical significance is indicated in the figure panels as follows: ns (not significant), *p* > 0.05; *p ≤ 0.05; **p ≤ 0.01; ***p ≤ 0.001; ****p ≤ 0.0001. In the graph, all data are represented as mean ± SD, except for Fig. 1j, k, Supplementary Fig. 1k, l, and Supplementary Fig. 3q data are represented as mean ± SEM.

## Supplementary information

Supplementary Figures and Tables

Transparent Peer Review file

## Data Availability

The numerical source data behind the graphs are available through the Goethe University Data Repository (GUDe)^85^. All plasmids newly generated in this study have been deposited and are available at Addgene. All other materials are available from the corresponding author upon reasonable request.

## Code availability

All source codes for pLLP cell count, volume, neuromast deposition, and Yap1 immunostaining analysis, as well as the rosette detector, are publicly available through the GUDe, 10.25716/gude.0m6t-db8z.
