# Supplementary material for: Two distinct modes of Vgll4-mediated Tead regulation control organ size in zebrafish
Source: Commun Biol. 2026 Apr 25;9:574. doi: 10.1038/s42003-026-10098-y (PMC13110368; doi:10.1038/s42003-026-10098-y)
Supplement: Supplementary file 1 — Supplementary Figures and Tables [file 42003_2026_10098_MOESM1_ESM.pdf]

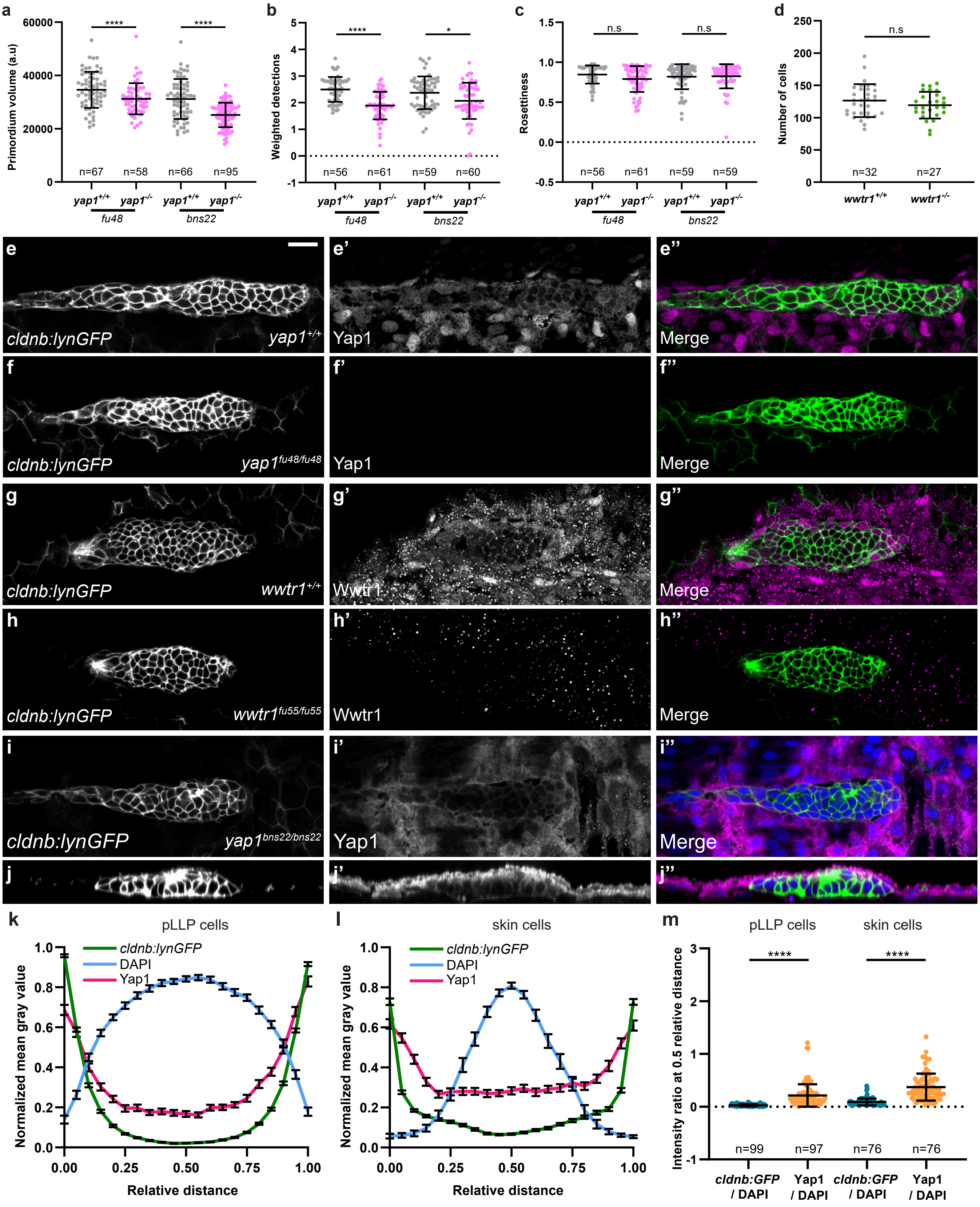

Supplementary Figure 1. Tead-binding is essential for Yap1-driven proliferation in the pLLP.

(a-c) Quantifications of total volume (a), rosette weighted detections (b) and rosettiness index (c) of the pLLP in in *yap1*$^{+/+}$ and *yap1*$^{-/-}$ siblings carrying the *fu48* or *bns22* alleles at 32 hpf (N ≥ 2 independent replicates). (d) Quantification of pLLP cell number in *wwtr1*$^{+/+}$ and *wwtr1*$^{fu55/fu55}$ embryos at 32 hpf (N = 2 independent replicates). (e-f'') Single Z plan of confocal Z-stacks showing Yap1 localization in control (e-e'') and *yap1*$^{fu48/fu48}$ 32 hpf embryos (f-f''). (g-h'') Single Z plan of confocal Z-stacks showing the localization pattern of Wwtr1 in control embryo (g-g'') as compared to that in *wwtr1*$^{fu55/fu55}$ (h-h''). (i-j'') Single Z plan of confocal Z-stacks (i-i'') and orthogonal views (j-j'') showing Yap1 localization in 32 hpf *yap1*$^{bns22/bns22}$ embryos. (k–l) Plot profile intensity across pLLP cells (k) and neighboring skin cells (l) in *yap1*$^{bns22/bns22}$ *cldnb:lynGFP* embryos at 32 hpf. Normalized mean gray values are shown for *cldnb:lynGFP* (membranes, green), DAPI (nuclei, blue), and Yap1 (magenta). (m) Quantification of normalized Yap1 and *cldnb:lynGFP* intensities relative to DAPI at 0.5 relative distance (N = 1 independent replicate). Data are presented as mean ± SD, except for panels k and l, data are mean ± SEM. Unpaired *t*-tests (Mann-Whitney) were conducted. Scale bars: 20 µm (e-j'').

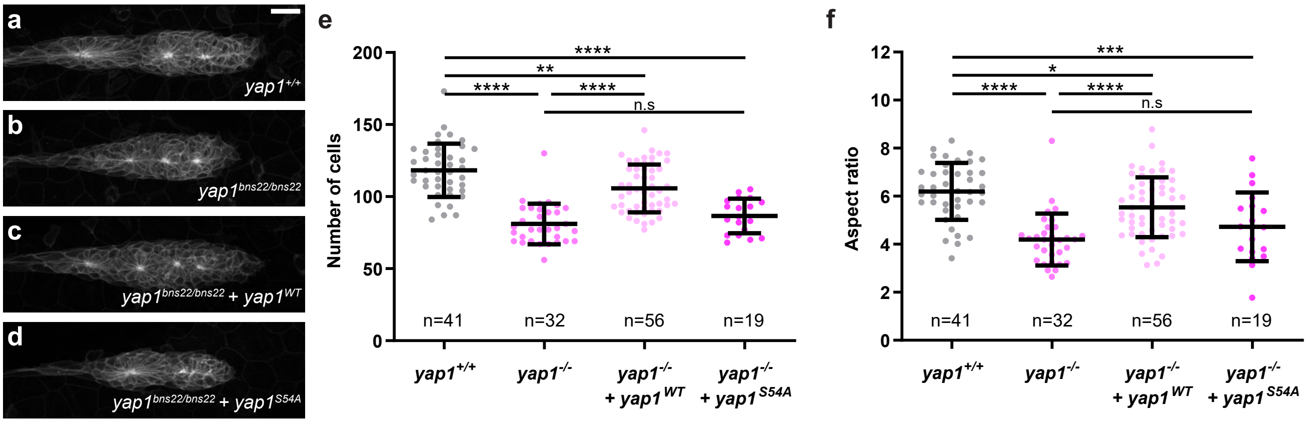

Supplementary Figure 2. A Tead-Binding-Deficient Yap1 mutant fails to rescue *yap1* loss-of-function phenotype.

(a-d) MIP of confocal Z-stacks showing the pLLP in uninjected *yap1*$^{+/+}$ (a), uninjected *yap1*$^{bns22/bns22}$ embryos (b), *yap1*$^{bns22/bns22}$ embryos injected with *yap1*-WT mRNA (c) and *yap1*$^{bns22/bns22}$ embryos injected with *yap1-S54A* mRNA (d). (e-f) Quantification of pLLP cell number (e) and aspect ratio (f) in each condition (N = 1 independent replicate). Data are presented as mean ± SD. Unpaired *t*-tests (Mann-Whitney) were conducted. Scale bar: 20 μm (a-d).

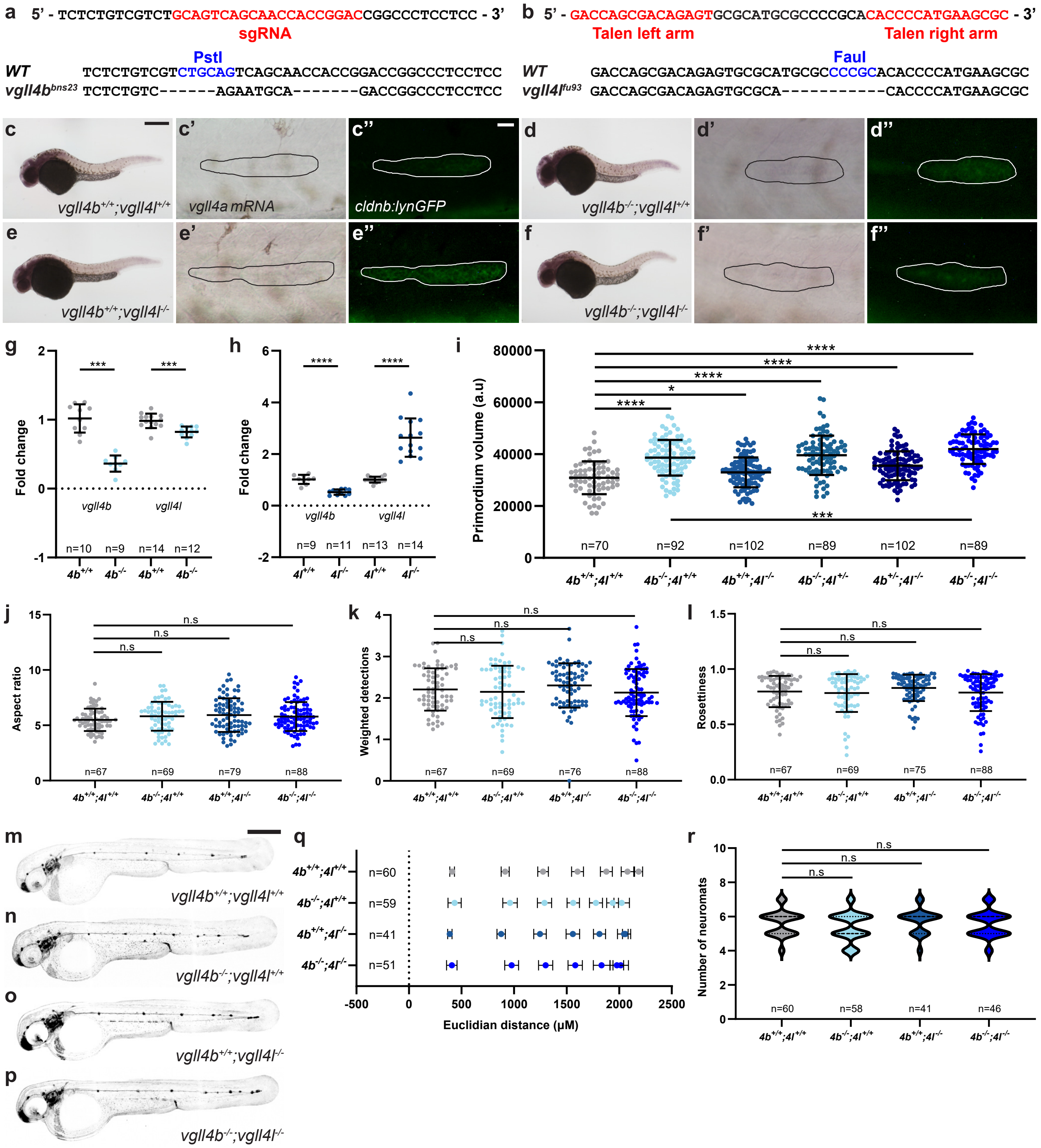

Supplementary Figure 3. Vgll4b and Vgll4l are required to limit the number of cells in the pLLP.

(a) CRISPR target sequence within the *vgll4b* exon2. (b) TALEN target sequence within the *vgll4l* exon2. (c-f'') Overview images of 32 hpf embryos stained with a *vgll4a* ISH probe in *vgll4b*$^{+/+}$;*vgll4l*$^{+/+}$ (c), *vgll4b*$^{-/-}$;*vgll4l*$^{+/+}$ (d), *vgll4b*$^{+/+}$;*vgll4l*$^{-/-}$ (e), and *vgll4b*$^{-/-}$;*vgll4l*$^{-/-}$ (f) embryos. Images of the pLLP at a higher magnification, stained with *vgll4a* ISH probe (c'-f') and an anti-GFP antibody (c''-f'') in the pLLP in the indicated genotypes.(g-h) *vgll4b* or *vgll4l* transcript levels measured by qPCR in *vgll4b*$^{-/-}$ (g) and *vgll4l*$^{-/-}$ (h) mutants at 32 hpf (N=2 independent replicates). (i-l) Quantifications of the total volume (i), aspect ratio (j), weighted detections (k), and rosettiness (l) of the pLLP in the indicated genotypes at 32 hpf (N≥4 independent replicates). (m-p) Overview images of 48 hpf *vgll4b*$^{+/+}$;*vgll4l*$^{+/+}$ (m), *vgll4b*$^{-/-}$;*vgll4l*$^{+/+}$ (n), *vgll4b*$^{+/+}$;*vgll4l*$^{-/-}$ (o), and *vgll4b*$^{-/-}$;*vgll4l*$^{-/-}$ (p) embryos. (q-r) Quantifications of neuromast deposition pattern (q) and total number of neuromasts (r) for each indicated genotype (N≥2 independent replicates). Data are presented as mean±SD, except for panel q, data are mean±SEM. Unpaired *t*-tests (Mann-Whitney) were conducted. Scale bar: 400 μm (c-f, m-p), 20 μm (c'-f'').

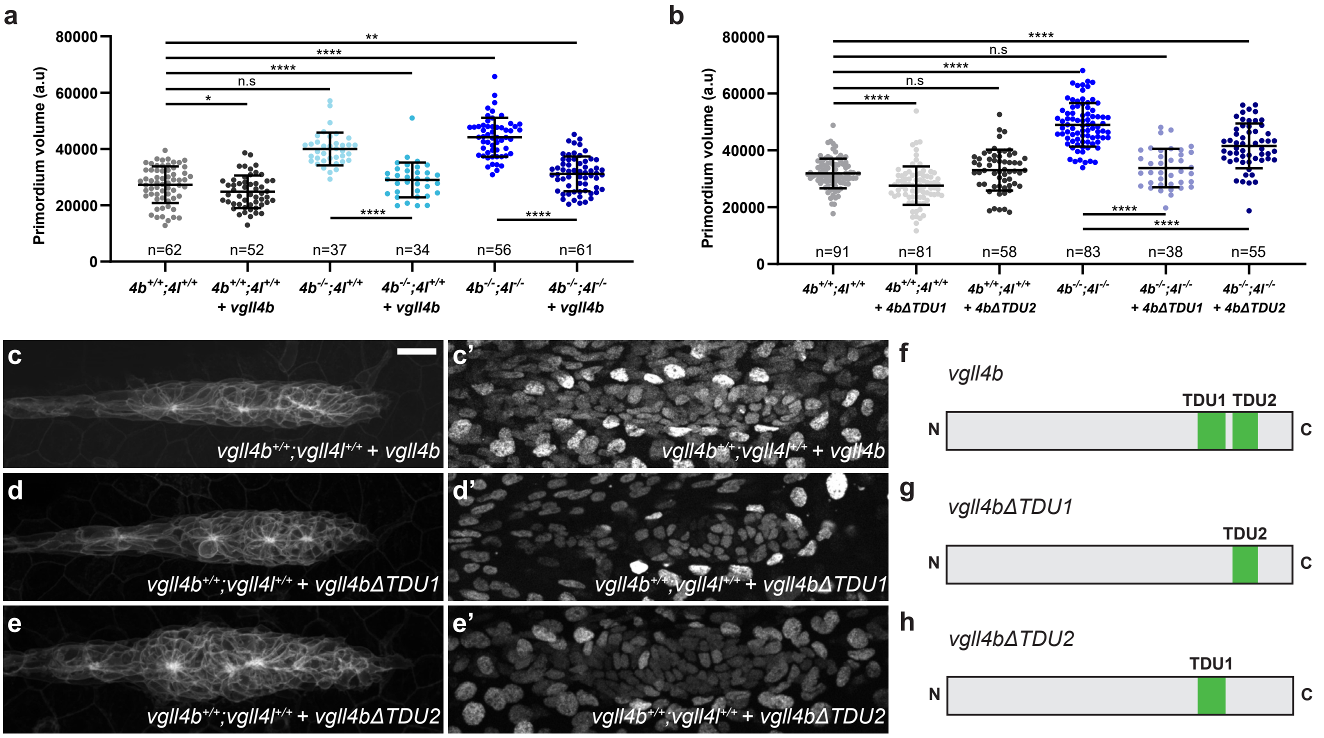

Supplementary Figure 4. Vgll4b and Vgll4l are sufficient to rescue the loss of Vgll4 activity in the pLLP, and Vgll4b TDU2 is required for this function.

(a-b) Quantification of pLLP volume in the indicated genotypes ($N \geq 2$ independent replicates). (c-e') MIP of confocal Z-stacks showing the pLLP (c-e) and the localization of *tRFP-vgll4b* (c'), *tRFP-vgll4bΔTDU1* (d') and *tRFP-vgll4bΔTDU2* (e') injected into *vgll4b*$^{+/+}$*;vgll4l*$^{+/+}$ embryos. (f-h) Schematic representations of full length Vgll4b (f) and the Vgll4b forms lacking TDU1 (g) or TDU2 (h). Data are presented as mean ± SD. Unpaired *t*-tests (Mann-Whitney) were conducted. Scale bar: 20 µm (c-e').

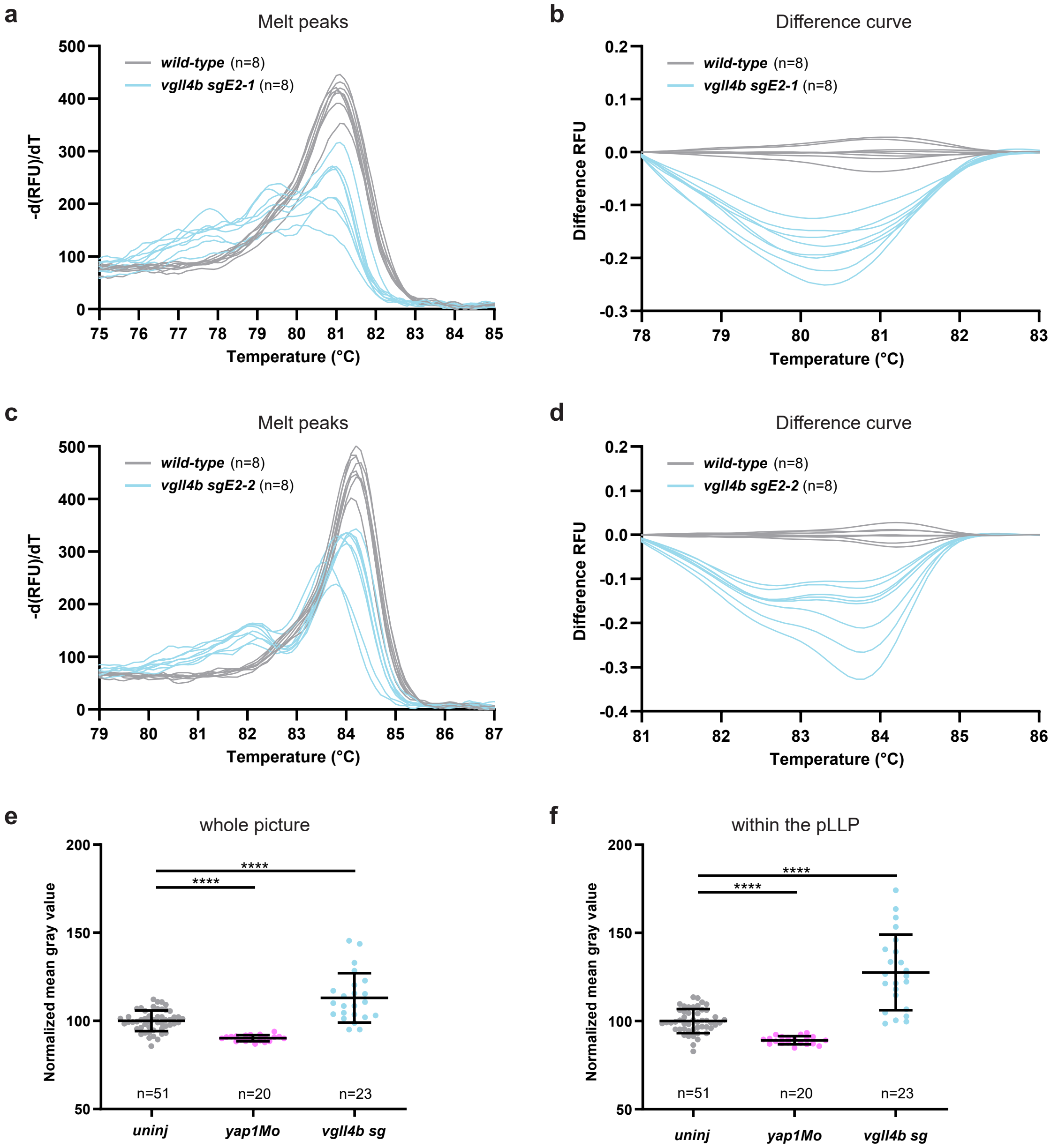

Supplementary Figure 5. Yap1 transcriptional activity is detected in the pLLP prior to the onset of migration.
(a-d) HRM melt peaks curves (a,c) and difference curves (b, d) of uninjected WT (grey curves) and embryos injected with Cas9 mRNA and either *vgll4b* sgRNA 1 or sgRNA 2, targeting exon 2, (blue curves) at 32 hpf. The blue curves are shifted and irregular compared to the grey WT curves, indicating the presence of INDELs (N = 1 independent replicate). (e,f) Quantification of normalized *GTIIC:d2EGFP* mean intensity measured on the average Z-Projection of the whole image (e) or within the pLLP on a single plane in the same embryos (f) (N ≥ 1 independent replicates). Data are presented as mean ± SD. Unpaired *t*-tests (Mann-Whitney) were conducted.

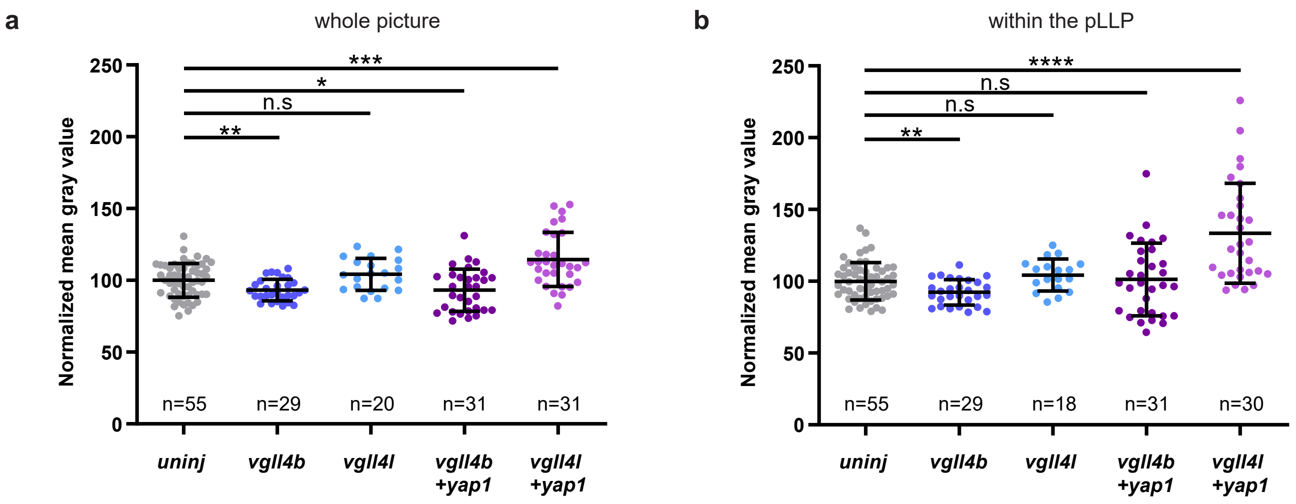

Supplementary Figure 6. Vgll4b suppresses Yap1-Tead activity more efficiently than Vgll4l in the pLLP.

(a, b) Quantification of normalized *GTIIC:d2EGFP* mean intensity measured on the average Z-Projection of the whole image (a) or within the pLLP on a single plane in the same embryos (b) (N ≥ 1 independent replicates). Data are presented as mean ± SD. Unpaired *t*-tests (Mann-Whitney) were conducted.

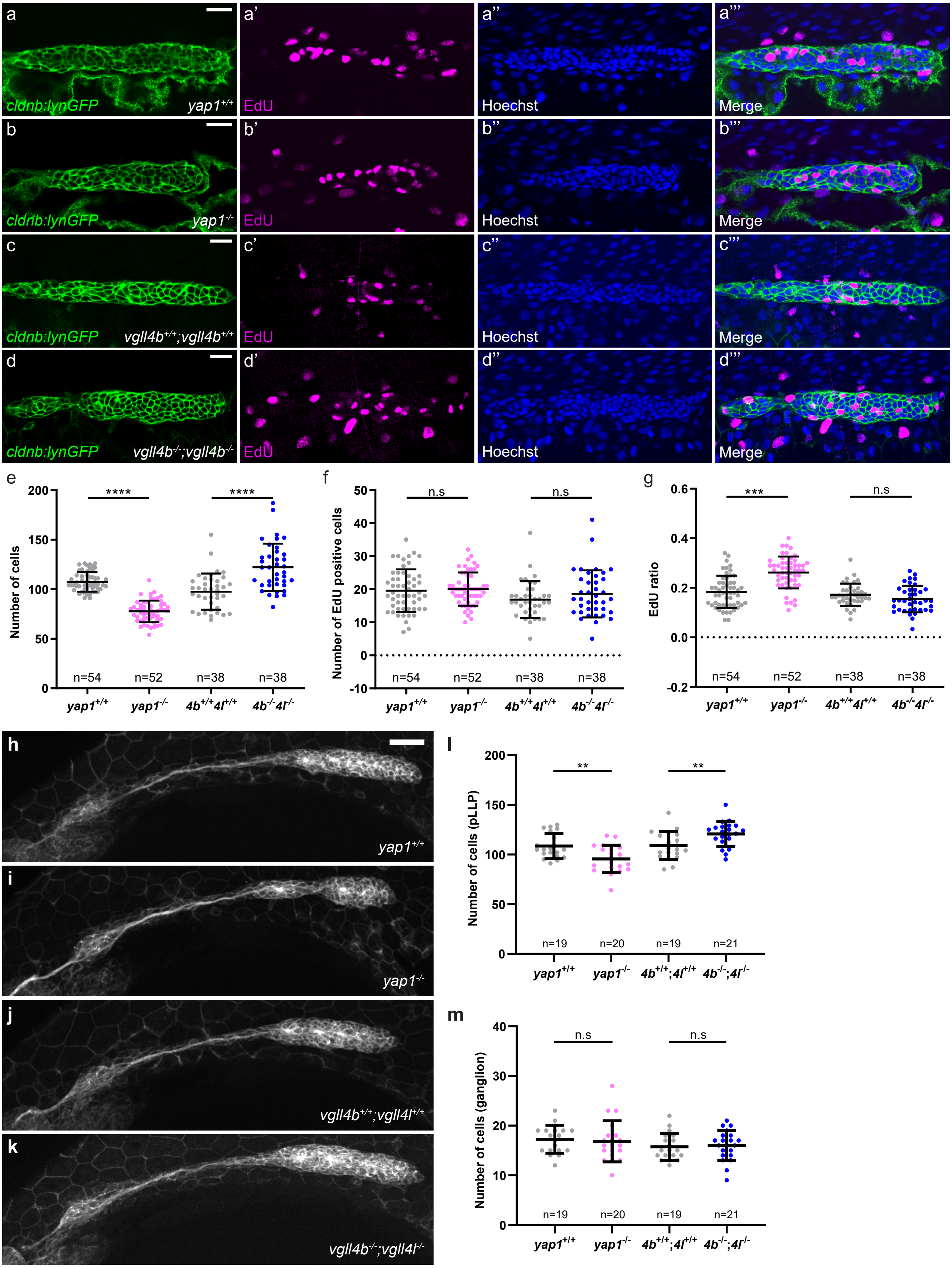

Supplementary Figure 7. ***yap1* and *vgll4b/l* are required for proper formation of the pLLP.**

(a-d) Single planes confocal images showing the amount of EdU stained nuclei in comparison to *cldnb:lynGFP* signal in $yap1^{+/+}$ (a-a’’’), $yap1^{-/-}$ (b-b’’’), $vgll4b^{+/+};vgll4l^{+/+}$ (c-c’’’), and $vgll4b^{-/-};vgll4l^{-/-}$ (d-d’’’) embryos at 32 hpf. (e-g) Quantification of pLLP cell number (e), EdU positive cell number (f) and ratio of EdU positive cell number to total pLLP cell number (g) in each condition (N = 2 independent replicates for *yap1*, N=1 for *vgll4*). (h-k) MIP of confocal Z-stacks showing the pLLP and the ganglion in $yap1^{+/+}$ (h), $yap1^{-/-}$ (i), $vgll4b^{+/+};vgll4l^{+/+}$ (j), and $vgll4b^{-/-};vgll4l^{-/-}$ (k) embryos at 26 hpf. (l-m) Quantification of pLLP (l) and ganglion cell number (m) in each condition (N = 1 independent replicate). Data are presented as mean ± SD. Unpaired *t*-tests (Mann-Whitney) were conducted. Scale bar: 20 µm (a-d’’’, h-k).

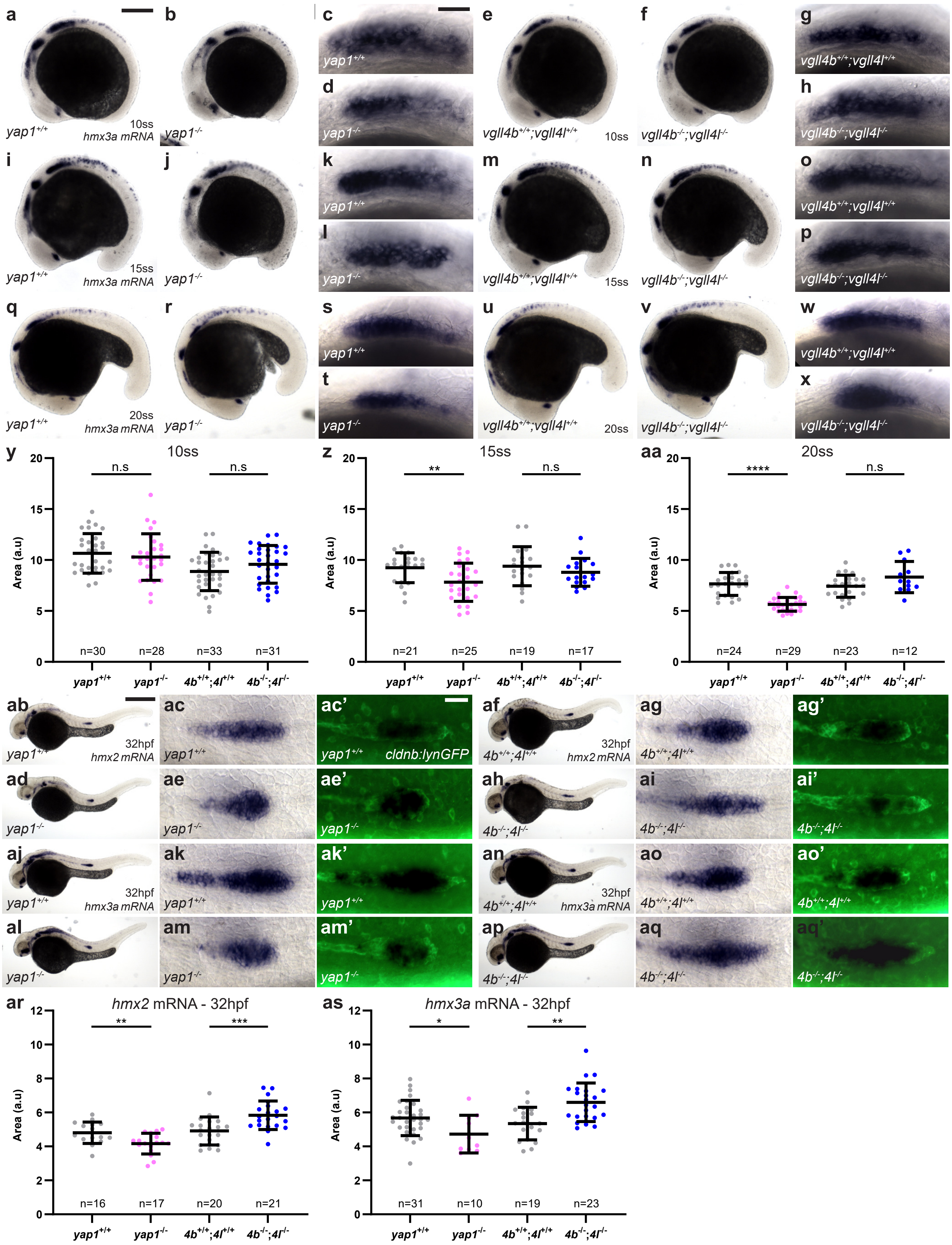

Supplementary Figure 8. pLLP size changes in *yap1* and *vgll4* mutants emerge between 15ss and 20ss.
(a-p) Overview (a, b, e, f, i, j, m, n, q, r, u, v) and higher magnification images (c, d, g, h, k, l, o, p, s, t, w, x) taken on a brightfield microscope of $yap1^{+/+}$ (a, c, i, k, q, s), $yap1^{-/-}$ (b, d, j, l, r, t), $vgll4b^{+/+};vgll4l^{+/+}$ (e, g, m, o, u, w), and $vgll4b^{-/-};vgll4l^{-/-}$ (f, h, n, p, v, x) embryos stained by ISH for *hmx3a* at 10ss (14 hpf) (a-h), 15ss (16.5 hpf) (i-p), and 20ss (19 hpf) (q-x). (y-aa) Quantifications of *hmx3a* stained area in each indicated group at 10ss (y), 15ss (z), and 20ss (aa) (N = 1 independent replicate). Overview images of 32 hpf embryos stained with *hmx2* (ab, ad, af, ah) or *hmx3a* (aj, al, an, ap) ISH probe in $yap1^{+/+}$ (ab, aj), $yap1^{-/-}$ (ad, al), $vgll4b^{+/+};vgll4l^{+/+}$ (af, an), and $vgll4b^{-/-};vgll4l^{-/-}$ (ah, ap) embryos. Images of the pLLP at a higher magnification, stained with *hmx2* (ac, ae, ag, ai) and *hmx3a* (ak, am, ao, aq) ISH probe and an anti-GFP antibody (ac', ae', ag', ai', ak', am', ao', aq') in the pLLP in the indicated genotypes. (ar, as) Quantifications of *hmx2a* (ar)and *hmx3a* (as) stained area in each indicated group at 32 hpf (N = 1 independent replicate). Data are presented as mean ± SD. Unpaired *t*-tests (Mann-Whitney) were conducted. Scale bar: 400 µm (a, b, e, f, i, j, m, n q, r, u, v, ab, ad, af, ah, aj, al, an, ap), 20 µm (c, d, g, h, k, l, o, p, s, t, w, x, ac, ac', ae, ae', ag, ag', ai, ai', ak, ak', am, am', ao, ao', aq, aq').

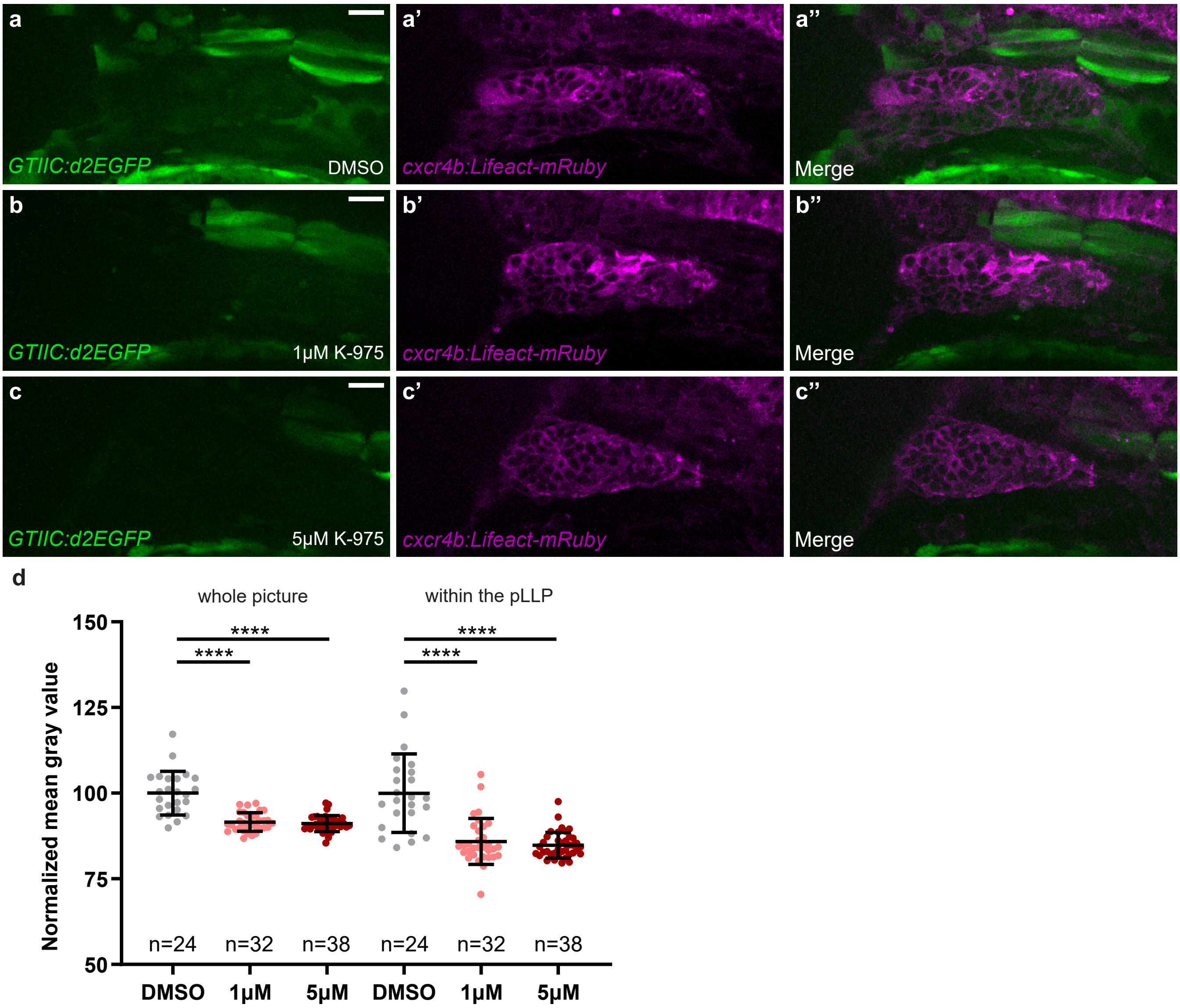

Supplementary Figure 9. Yap1 activity is required between 10ss and 20ss for proper pLLP formation.

(a-c’’) Single-plane confocal images of the pLLP in 20 hpf embryos treated at 30% epiboly with DMSO (a), 1μM (b) or 5μM K-975 (c) showing *GTIIC:d2EGFP* (a, b, c), *cxcr4b:Lifeact-mRuby* (a’, b’, c’) and merged channels (a’’, b’’, c’’). (d) Mean grey value measured either on the whole image or within the pLLP on a single plane in the GFP channel in the same embryos for each condition (N = 2 independent replicates). Data are presented as mean ± SD. Unpaired *t*-tests (Mann-Whitney) were conducted. Scale bar: 20 µm (a-c’’).

Table S1 – primers used in this study

Procedure	Gene / Construct	Direction	Primer sequence (5' to 3')
**TALEN**	*vgll4l*	*fwd*	ACCTTTTCACAAGCAAGCGA
		rev	AACATCCGCTGCGTAAAACA
**CLONING cDNA in pCS2 / pCS2-FP**	*tRFP-vgll4b*	*fwd*	AATACGAATTCGATGCTTTTTACCAAAATGGACCT
		rev	AGAGGCTCGAGTCAAGACACCAGGGACGG
	vgll4l	*fwd*	AATACGAATTCGATGGCGGTCACTAATTTCCAC
		rev	AGAGGCTCGAGTCATTTATCAGACCAGAAGTTG
	tRFP-vgll4bΔTDU1	*fwd*	CGAACACCGCCTGTAAGGAGCCCGAGCCCGTC
		rev	GCTCGGGCTCCTTACAGGCGGTGTTCGAGTTG
	tRFP-vgll4bΔTDU2	*fwd*	CAAACTCCGTGTCCATCCTGCAGATCAAGGC
		rev	CTTGATCTGCAGGATGGACACGGAGTTTGTG
	GFP-yap1	*fwd*	AAGACGAATTCGATGGATCCGAACCAGCACA
		rev	AGAGGCTCGAGCTATAGCCAGGTTAGAAAGTTCTCCTT
	GFP-yap1-S54A	*fwd*	CTGCCAGACGCTTTCTTCAC
		rev	GTGAAGAAAGCGTCTGGCAG
**CLONING cDNA in pGEM-T**	*vgll4a* ISH probe	*fwd*	AATGCAGCATTCAGGGTCAGG
		rev	AATGCAGCATTCAGGGTCAGG
	vgll4b ISH probe	*fwd*	CACCTGTGCTCCAGCTAACA
		rev	AACTGACACGACCTCCATCC
	vgll4l ISH probe	*fwd*	TGATCAAGAAACCACGACCA
		rev	AGCTTCTCCAACGACGCTTA
	hmx2 ISH probe	*fwd*	GAGGACTCGGGTGACTGCTA
		rev	ACGTAGACTCGTTTAATTGCTGC
	hmx3a ISH probe	*fwd*	TTTGCGTTACCGGCTTACCT
		rev	GTCGGGCTCAAATGCATGATA
**GENOTYPING**	*vgll4b*bns23*	*fwd*	GCGTACGCGTCCCTTAATTGG
		rev	GGACACAAGCTGTCGGATGC
	*vgll4l*fu93*	*fwd*	ACATGACCAGCGACAGAGTG
		rev	GACGGTACACAGTGACAGGA
	*yap1*bns22*	*fwd*	GCGCGTTTCCACATTTATTT
		rev	GTGACACTGCAGGCTGAAAG
**RT-PCR**	*vgll4b*	*fwd*	TCACCTGTGCTCCAGCTAAC
		rev	TCCTCGATCACAGGGTCACA
	vgll4l	*fwd*	GCTGCAATCACTCAACTGCTGAA
		rev	ATGGACGTGGCTCTGTGGTAG
	yap1	*fwd*	AATGACCAGACGACCACCTG
		rev	ATAGCTTGTTCCCACCCGTC
	rpl13	*fwd*	TAAGGACGGAGTGAACAACCA
		rev	CTTACGTCTGCGGATCTTTCTG
**HRM**	*vgll4b* – Exon2-1	*fwd*	GACGACATCGACTGCAACAG
		rev	TGCACCCTGAATGAGACACA
	vgll4b – Exon2-2	*fwd*	TGAGAGGCGAGTCCAGAATG
		rev	ATGTCGTCGTCTGCTTGCTC

Table S2 – plasmids generated for this study

Procedure	Plasmids obtained	Addgene ID
**CLONING cDNA in pCS2 / pCS2-FP**	pCS2-tRFP-vgll4b	254506
	pCS2-vgll4l	254507
	pCS2-tRFP-vgll4bΔTDU1	254508
	pCS2-tRFP-vgll4bΔTDU2	254509
	pCS2-GFP-yap1	254510
	pCS2-GFP-yap1S54A	254511
	pCS2-tBFP-yap1	254512
**CLONING cDNA in pGEM-T**	pGEM-T-vgll4a	254513
	pGEM-T-vgll4b	254514
	pGEM-T-vgll4l	254515
	pGEM-T-hmx2	254516
	pGEM-T-hmx3a	254517
