# Supplementary material for: Two distinct modes of Vgll4-mediated Tead regulation control organ size in zebrafish
Source: Commun Biol. 2026 Apr 25;9:574. doi: 10.1038/s42003-026-10098-y (PMC13110368; doi:10.1038/s42003-026-10098-y)
Supplement: Supplementary file 2 — Transparent Peer Review file [file 42003_2026_10098_MOESM2_ESM.pdf]

nature portfolio

Peer Review File

Two distinct modes of Vgll4-mediated Tead regulation control organ size in zebrafish

Corresponding Author: Professor Virginie Lecaudey

This manuscript has been previously reviewed at another journal. This document only contains information relating to versions considered at Communications Biology.

This manuscript is considered suitable for publication without peer review at Communications Biology.

Open Access This Peer Review File is licensed under a Creative Commons Attribution 4.0 International License, which permits use, sharing, adaptation, distribution and reproduction in any medium or format, as long as you give appropriate credit to the original author(s) and the source, provide a link to the Creative Commons license, and indicate if changes were made.
In cases where reviewers are anonymous, credit should be given to 'Anonymous Referee' and the source.
The images or other third party material in this Peer Review File are included in the article's Creative Commons license, unless indicated otherwise in a credit line to the material. If material is not included in the article's Creative Commons license and your intended use is not permitted by statutory regulation or exceeds the permitted use, you will need to obtain permission directly from the copyright holder.
To view a copy of this license, visit https://creativecommons.org/licenses/by/4.0/
